# Reversible Deactivation Radical Polymerization in Biomass-Derived Solvents: Sustainable Approach in Polymer Chemistry

**DOI:** 10.1021/acspolymersau.5c00114

**Published:** 2025-10-10

**Authors:** Agata Hochół, Izabela Zaborniak, Magdalena Bednarenko, Alessandro Pellis, Krzysztof Matyjaszewski, Paweł Chmielarz

**Affiliations:** † Department of Physical Chemistry, Faculty of Chemistry, 69696 Rzeszow University of Technology, Al. Powstańców Warszawy 6, 35-959 Rzeszów, Poland; ‡ Doctoral School of Rzeszów University of Technology, Al. Powstańców Warszawy 12, 35-959 Rzeszów, Poland; § Department of Chemistry and Industrial Chemistry, 9302 University of Genova, Via Dodecaneso 31, 16146 Genova, Italy; ∥ Department of Chemistry, 6612 Carnegie Mellon University, 4400 Fifth Avenue, Pittsburgh, Pennsylvania 15213, United States

**Keywords:** biomass-derived solvents, green chemistry, RDRP, polymer synthesis, solvent effect, lignocellulosic biomass, essential oils, vegetable oils

## Abstract

Motivated by growing concerns over the impact of conventional organic solvents on humans and their environmental safety, this review highlights recent advancements in the application of renewable resource-derived solvents in reversible deactivation radical polymerization (RDRP) techniques, with a focus on their effectiveness and potential benefits. The review begins with a concise overview of the importance of environmentally friendly solvents, outlining the specific requirements associated with their use. It then details the key parameters used to classify and evaluate solvents. Subsequently, the review examines the role and influence of solvents in various RDRP techniques, with particular attention to green solvents, especially those derived from biomass, that are gaining increasing attention in polymer synthesis. Three major classes of biomass-derived solvents are discussed: (1) lignocellulosic biomass, (2) essential oils, and (3) vegetable oils. These sustainable alternatives address both the depletion of fossil resources and environmental concerns associated with traditional solvents. The review highlights the promising outcomes achieved using these biobased solvents, demonstrating their potential to enhance the sustainability and environmental compatibility of controlled polymer synthesis.

## Graphical abstract

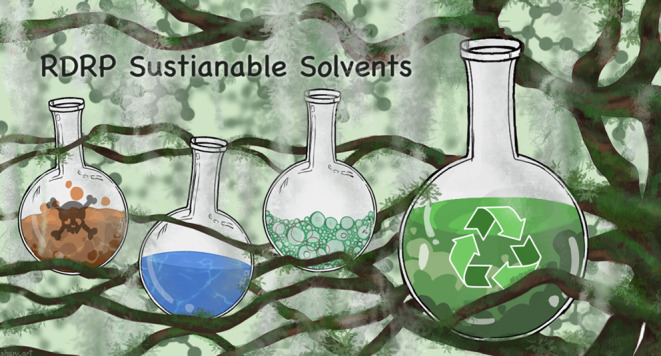

## 1 Introduction

In response to growing public awareness of environmental and human health concerns, there is an urgent need to adopt more sustainable and eco-friendly practices, particularly regarding solvents, which are widely used across numerous fields, including broadly defined chemistry and engineering. Solvents serve critical functions: they dissolve reagents, facilitate their interaction, enable heat transfer, and significantly influence the success of chemical reactions. Key solvent properties such as polarity, dipole moment, and their ability to coordinate with metal centers are essential in many applications, including organic syntheses,^1,2^ purification techniques, chromatography or liquid–liquid extraction,^3,4^ analytical methodologies,^5^ as well as catalysis^6,7^ and polymer synthesis.^8^ Beyond industrial applications, solvents are heavily used in household products and consumer goods. Figure 1 illustrates solvent consumption across various sectors. This widespread use necessitates a thorough reassessment of the potential health and environmental risks associated with solvent exposure. Although often viewed as a concern limited to chemists or those working directly with chemicals, the adverse effects of solvent use reach far beyond the laboratory. The harmful impact on both human health and the environment is a global concern that demands immediate and sustained action.

**Figure 1:**
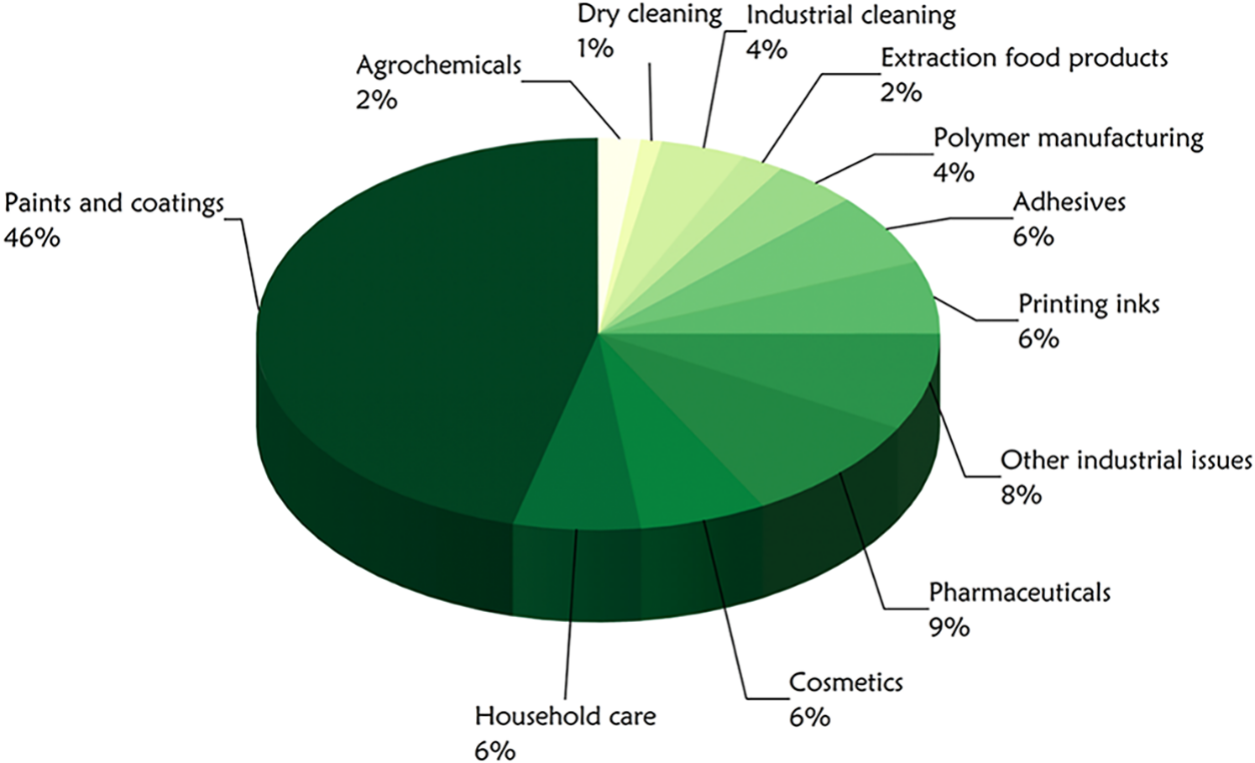
Average consumption of solvents in different industries. Adapted with permission from ref 4. Copyright 2018 Springer Nature.

The primary objective of future research in this area is to reduce the environmental impact of solvents. This can be achieved by replacing toxic fossil fuel-derived solvents with biobased alternatives. Naturally derived solvents can be sustainably produced from biomass through fermentation, chemical transformation, or by utilizing postprocessing waste. Renewable sources primarily include agricultural crops rich in lignocellulose and carbohydrates, such as corn, beets, wheat, as well as wood waste, scrap paper, aquatic biomass (e.g., microalgae), and plant oils or organic residues that have long been regarded as waste. The versatility of these sources allows for the production of solvents with a diverse range of properties, including alcohols, esters, ethers, carboxylic acids, or ketones, which can serve as effective replacements for numerous toxic solvents.^3,4^

This raises an important question: what qualifies a solvent as truly environmentally friendly, or a “green solvent”? It is important to note that not all naturally derived solvents are inherently green; there are a few exceptions. Nevertheless, biobased solvents are generally more environmentally friendly than petroleum-based ones.^9^ Before adopting a solvent, multiple factors must be carefully evaluated. It is of paramount importance to ascertain how the solvent is manufactured and whether it necessitates intricate, costly, and energy-intensive purification procedures. Solvent assessment methods can be broadly classified into two categories: (1) general factors, i.e., manufacturing energy, total energy consumption, and the overall impact on human health and the environment, (2) application-specific parameters – factors that relate to the solvent’s suitability for particular uses.^10^ To effectively replace toxic solvents, it is essential to identify green alternatives that offer comparable physical and chemical properties, enabling similar performance in practical applications. Furthermore, the availability and biodegradability of potential biobased solvents must be considered. A summary of the key criteria for green solvents is provided in Figure 2.

**Figure 2:**
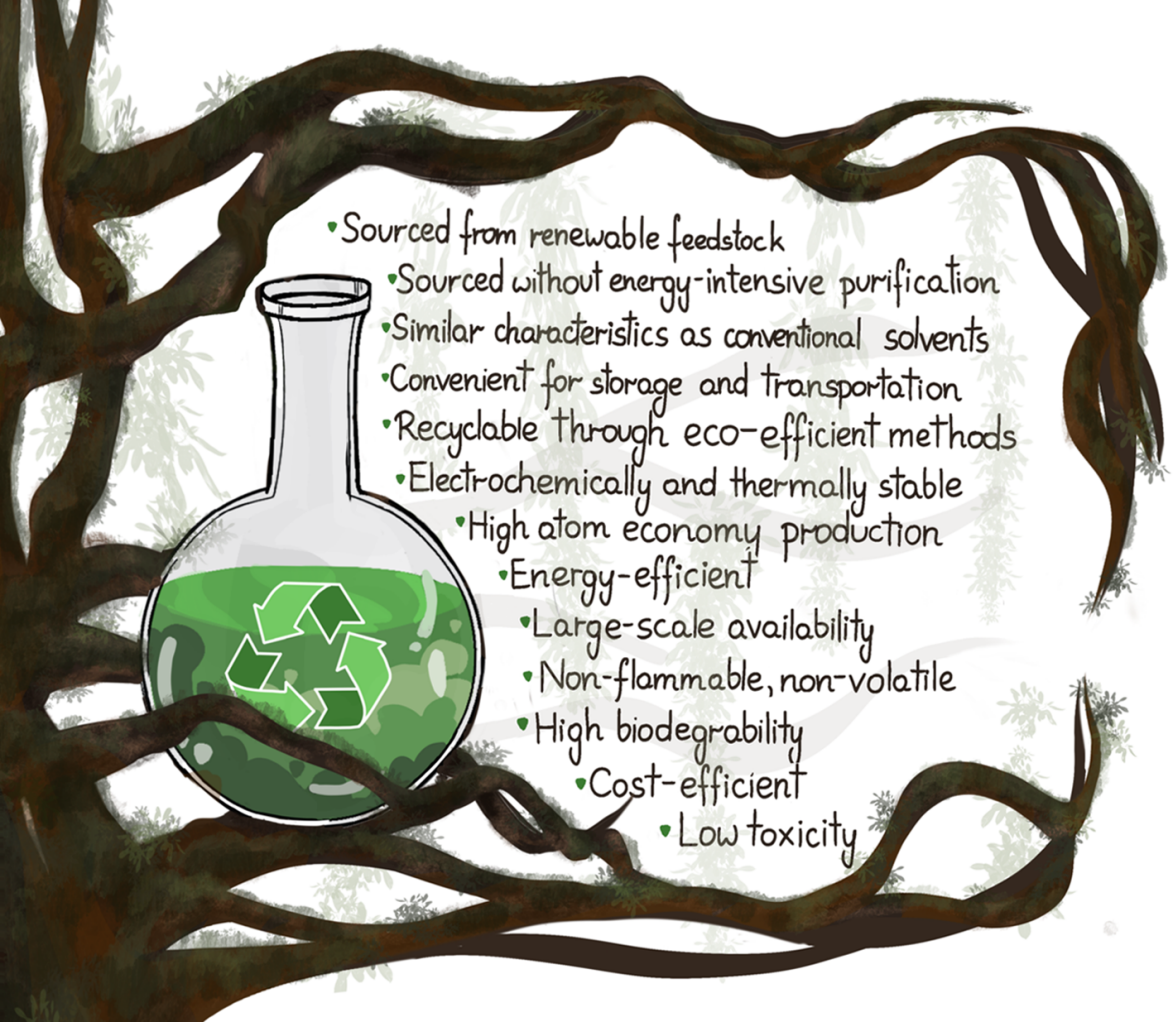
Green solvents requirements.

A recent review^2^ provides a thorough and detailed discussion encompassing potential solvent alternatives, their production methods, and the wide range of applications in which they are utilized. The authors examine greener, safer, and more sustainable alternative solvents derived from biorenewable resources, emphasizing their potential for various applications in synthetic organic chemistry. However, while green solvents are derived from natural sources, it is important to recognize that biobased compounds can still undergo environmentally harmful production processes. Conversely, advancements are being made to reduce the environmental impact of processes involving petroleum-based molecules.

To ensure that a proposed solvent alternative is genuinely more sustainable than the original, conducting a life cycle analysis (LCA) is essential. An LCA provides a comprehensive, objective assessment of a solvent’s environmental footprint from production to disposal. To fully assess its impact, the solvent’s life cycle should be evaluated from raw material acquisition and production, through manufacturing, distribution, and use, to final disposal or recycling. Each stage is crucial for understanding and minimizing both the environmental and economic impacts of the solvent. In some cases, the environmental burden from raw material extraction, solvent production, and transportation can exceed the emissions generated during solvent use. Therefore, an accurate assessment of the overall environmental impact must account not only for direct emissions but also for the total resource consumption and emissions produced throughout the entire production process of each chemical involved.^11,12^ Therefore, while the shift toward biobased solvents is promising, it must be approached with caution. The goal is to develop solvents that are not only biobased but also environmentally responsible – green, but not at any cost.^9,13^

In light of this consideration, the primary objective of this study is to provide a comprehensive overview of current research and significant advances achieved in the use of biomass-derived solvents in polymer synthesis, with particular focus on reversible deactivation radical polymerization (RDRP) techniques. Since the development of RDRP techniques, considerable progress has been made to reduce their environmental impact. A key milestone in atom transfer radical polymerization (ATRP) was the introduction of an additional redox cycle to regenerate the active form of the copper catalyst, thereby reducing the required catalyst concentration to just tens of ppm.^14,15^ Building on this progress, research has shifted toward employing eco-friendly reducing agents such as ascorbic acid, as well as commonly available substances such as honey,^16,17^ vitamins,^18–20^ or condiments.^21^ Common beverages, including red wine,^22^ flavored alcohols,^23,24^ and coffee,^25^ were also examined for their unique chemical compositions that may facilitate reduction reactions. Furthermore, the sustainability of RDRP processes has been enhanced through the application of UV^26,27^ and visible^28,29^ radiation as well as electric current^30^ to regenerate the catalyst. The application of UV/Vis radiation facilitated the elimination of metal catalysts in metal-free ATRP through the employment of organic photoreducible catalysts,^31–34^ especially of natural origin, like curcumin^21^ or riboflavin.^18,35^ Within the frameworks of ATRP and reversible addition–fragmentation chain-transfer (RAFT), the implementation of green chemistry principles has primarily focused on enhancing energy efficiency and facilitating the polymerization of monomers derived from renewable resources.^29,36,37^

Despite these advances, conventional ATRP and RAFT methods still rely on volatile organic solvents, which undermine their environmental sustainability. Solvents, typically comprising around 50% of the reaction mixture volume (and in some cases up to 70%), are a significant component of the polymerization system. Consequently, recent studies emphasize that assessing sustainability in RDRP must include the choice of an environmentally friendly solvent, alongside the source of monomers and catalysts.^15,36,37^

These developments highlight a clear trend: aligning ATRP and RAFT techniques with the principles of green chemistry by replacing hazardous solvents with renewable, biomass-derived alternatives. This review explores the emerging role of green solvents, including cellulose-derived ketones and esters, terpene-based solvents, and others, in advancing the sustainability of RDRP.

## 2 Parameters for Solvent Classification and Assessment

The development of new biobased solvents has created a need for universal characterization parameters, predictive tools, and selection guidelines to facilitate the replacement of toxic solvents in specific applications. Since the pharmaceutical industry generates the majority of chemical waste, with solvents representing the largest share, many pharmaceutical companies have developed solvent classification guides based on green chemistry principles. For instance, Pfizer was among the first to develop a solvent selection guide, classifying solvents into three categories using a color-coded method.^4^ Similarly, GlaxoSmithKline (GSK) developed a solvent selection guide expanded with a life cycle assessment.^38^ Solvents are evaluated based on the following parameters:

- Waste management: including scores for incineration, recycling, biotreatment, and volatile organic compound (VOC) emissions
- Environmental impact focusing on effects on the air and water environment
- Health considerations: including health hazard ratings and exposure potential
- Safety: including assessments of flammability, explosion potential, reactivity, and chemical stability
- Life cycle analysis: evaluating the overall environmental burden throughout the solvent’s life cycle.

Despite the availability of solvent selection guides developed by pharmaceutical corporations, more universal and precise tools for solvent evaluation are the Kamlet–Abboud–Taft (KAT) parameters and the Hansen solubility parameters (HSP).^39^ These parameters enable a direct comparison with conventional toxic solvents, evaluation of their compatibility with materials and products, and ultimately support the identification and implementation of more sustainable alternatives. The physicochemical properties, solubility parameters, and toxicity data for biomass-derived solvents are summarized in Table 1. KAT parameters are determined by evaluating the effect of a solvent on a solute. These parameters describe the polarity of the solvent to free energy relationships, selectivity of product formation, reaction equilibria, redox potentials, reaction rate constants, as well as spectroscopic behavior. The KAT parameters are described by α – hydrogen bond donating ability; β – hydrogen bond accepting ability; and π* – dipolarity and polarizability. Each parameter is expressed on a normalized scale from 0 to 1.^40–42^ HSPs, on the other hand, are theoretical descriptors, calculated by experimental data sets or predicted by different calculation methods. The Hansen solubility parameters are widely used to characterize a broad range of chemical entities, including solvents, polymers, nanoparticles, drugs, salts, CO_2_, and even biological systems. The parameters are based on three key types of intermolecular interactions: δ_D_ – dispersion forces, δ_P_ – polar (dipole–dipole) interactions, and δ_H_ – hydrogen bonding ability expressed in units of MPa^1/2^.^9,43,44^ These three parameters collectively constitute the Hansen total solubility parameter (δ_total_), which represents the cohesive energy density of a substance. The term “cohesive energy” refers to the energy required to separate molecules in the liquid phase from the gas phase. However, since this energy is highly dependent on molecular size (larger molecules require more energy), it is normalized by dividing the cohesive energy (*E*) by the molar volume (*V*). The resulting cohesion energy is represented by the equation δ_total_^2^ = *E*/*V*.^43^ This formulation allows for meaningful comparison of solubility behavior across molecules of varying sizes.^45^

**Table 1:**
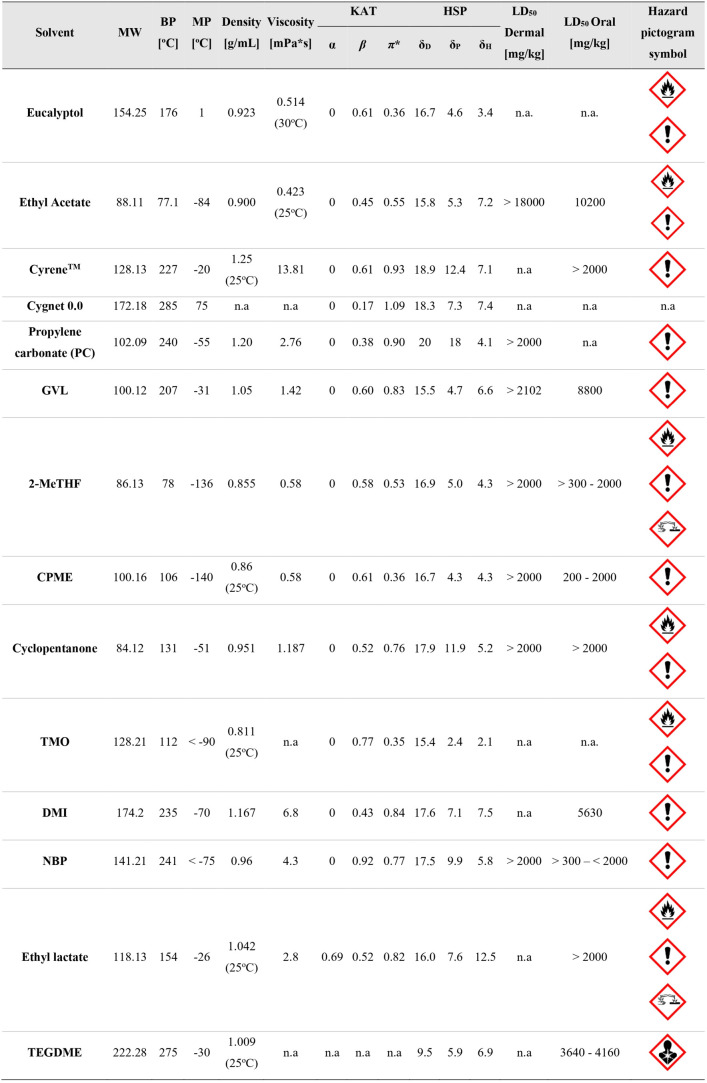
Physicochemical Properties, Solubility Parameters, and Toxicity of Biomass-Derived Solvents^a^

a Data gathered from various sources,^2,50–59^ and safety data sheets from chemical producers; n.a—data not available.

Hansen solubility parameters have proven to be more effective than nonspecific concepts such as “polar” versus “nonpolar” or “hydrophilic” versus “hydrophobic”. Unfortunately, many studies continue to rely on simplistic, one-dimensional measures like Log *P* – the octanol–water coefficient – to explain complex behaviors. In contrast, HSPs provide a multidimensional representation of a substance’s solubility characteristics through three distinct parameters, which Log *P* alone cannot capture. Thus, HSPs offer not only deeper insight into solubility phenomena but also strong predictive capabilities. HSPs are particularly useful for selecting appropriate solvents for the dissolution of diverse chemical compounds, following the principles of “*like dissolves like*”, when the solubility parameters of the solvent and the solute are similar. In practice, solvents are plotted in a three-dimensional (3D) coordinate system known as HSP space, with each solvent represented by a point defined by three parameters (δ_D_, δ_P,_ δ_H_). Importantly, perfect matching of HSP values is not required to achieve complete solubility. Instead, solvent efficacy can be estimated by examining the relative position of solvent and solute points in HSP space. The most effective solvents typically fall within a spherical region centered around the solute’s HSP values. Solvents located within this sphere are considered good solvents, while those outside the sphere tend to be less effective (see examples in Figure 3).^43^

**Figure 3:**
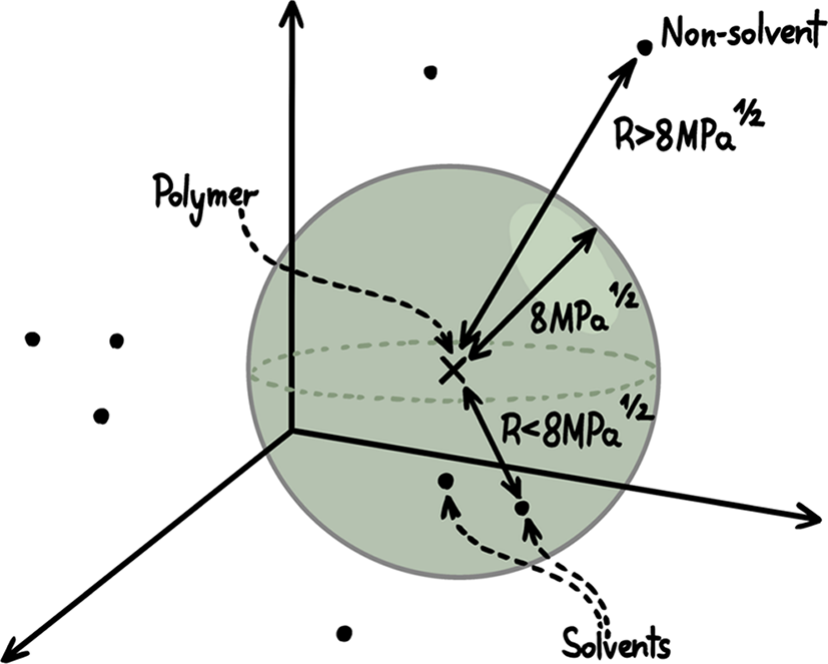
Hansen space representation of solvents and nonsolvents falls within or outside a sphere of radius *R* = 8 MPa^1/2^ centered on the polymer, respectively. Adapted with permission from ref 46. Copyright 2019 American Chemical Society.

The primary challenge associated with HSPs lies in accurately estimating their values, which remains an area of ongoing research. Although HSPs are highly dependent on molecule structure, most predictive methods currently available lack sufficient precision, especially when dealing with complex intergroup interactions. So far, the optimal methodology for predicting HSPs, especially for solubility in nonconventional solvents, is the Yamamoto-molecule breaking (Y-MB) method. The Y-MB method automates the fragmentation of molecules into subgroups and utilizes a comprehensive HSP database integrated with a neural network model. This combination allows for the fitting of group interactions based on the relative strengths of neural interconnections, thereby optimizing the predictive power of HSP. In addition to HSP values, the Y-MB method can also estimate other physicochemical parameters, such as boiling point, melting point, density, viscosity, and vapor pressure.^9^ It is worth mentioning that when a single substitute solvent is either unavailable or unprofitable, the combination of multiple solvents into a blend can provide a more suitable and efficient alternative. The use of HSP enables rapid optimization of solvent mixtures by comparing their solubility parameters, eliminating the need for time-consuming trial-and-error methods. Beyond solvent substitution, HSPs contribute to improving the recyclability of products by promoting alterations in base polymers and other formulations. Furthermore, the HSP method is advantageous in health, safety, and environmental (HSE) assessments by offering insight into solubility pathways of chemicals and the associated risks of chemical exposure. As the use of HSPs in HSE analyses becomes more widespread, it serves to reinforce the objective of minimizing the potential harms associated with chemical usage. Through systematic parameter assessment and blend optimization, the HSP approach promotes both sustainable development and safer chemical practices.^43,47^

Nevertheless, it is important to note that HSP and KAT parameters primarily capture similarities in the physicochemical properties of solvents. Although they are undoubtedly valuable tools for identifying alternatives to toxic solvents, they do not provide a holistic evaluation of environmental and human health impacts. For this purpose, the essential methodology is LCA.^11,48^ It enables the quantification and integration of the overall footprint, covering a broad range of impact categories that can be classified into midpoint and end point indicators. Midpoint indicators are problem-oriented, focusing on specific environmental mechanisms.^12,48^ These include:

(1) Cumulative energy demand
(2) Air acidification
(3) Biodiversity
(4) Ecotoxicity
(5) Human toxicity
(6) Eutrophication (marine and freshwater)
(7) Global warming
(8) Ionizing radiation
(9) Land use
(10) Ozone depletion
(11) Particulate matter
(12) Photochemical ozone creation
(13) Resource depletion
(14) Water scarcity.

End point indicators, by contrast, are damage-oriented and address broader areas of protection.^12,48^ These include

(1) Human health
(2) Ecosystem quality
(3) Natural resources.

Given the wide range of parameters that influence solvent sustainability, the use of multiple characterization models and data sources are required (see the comprehensive LCA handbook in ref 49). A persistent limitation in many solvent selection approaches is the lack of LCA data, particularly for emerging solvents, especially those derived from biomass. Furthermore, when LCA is applied, its scope is often limited – commonly excluding the end-of-life stage, and it frequently overlooks trade-offs associated with plant harvesting and the extraction of key substances for solvent production and purification.^11,12,48^ These trade-offs may include agricultural impacts, such as eutrophication caused by pesticides and chemical fertilizers, as well as land occupation. From a sustainable chemistry perspective, this consideration is critical, since the use of renewable feedstock alone does not inherently guarantee a “greener” solution. In fact, upstream production and processing of nonfossil resources can sometimes generate higher greenhouse gas emissions or, while reducing gas emissions, introduce greater human toxicity.^48^ Consequently, the identification of genuinely green and sustainable solvents remains complex and often contested. To avoid burden shifting, evaluations must integrate multiple environmental criteria within a full life cycle perspective.

## 3 Biomass-Derived Solvents in RDRP

As RDRP techniques acquire increasing attention, it is crucial to consider key factors that support the development of more sustainable chemical processes. Among the various RDRP methods, ATRP and RAFT polymerization stand out as the most commonly used, offering extensive possibilities in the field of controlled polymer synthesis. The remarkable benefit of employing RDRP techniques is the ability to precisely manipulate and control not only the molecular weight and molecular weight distribution (MWD) of the resulting polymers but also their architecture. Furthermore, maintaining the functionality at the ends of the polymer chains opens the possibility of synthesizing block copolymers with a predetermined arrangement of blocks and facilitates the incorporation of specific functionalities at the chain ends. This level of control represents a significant improvement over conventional radical polymerization methods.^37,60,61^ To achieve the desired level of control over the process, several important factors must be carefully considered and addressed. Beyond the choice of monomers, catalytic complexes, and initiators, the reaction medium plays a vital role in regulating polymerization behavior. Selecting an appropriate solvent is essential to ensure the solubility of all reagents in the reaction mixture and to fine-tune the system’s viscosity. Reagent diffusion limitations can significantly influence radical propagation and termination steps, potentially leading to a loss of control over the polymerization process. Moreover, understanding the interaction between the solvent and the other components in the system is crucial to minimize the potential for side effects that could lead to uncontrolled polymerization.^62–66^ Ultimately, solvent selection is often constrained by the chemical nature of the monomers, catalysts, and the targeted polymer structure.

The primary consideration in ATRP is the influence of the reaction medium, which determines the yield of polymerization. The choice of solvent can alter reaction kinetics by affecting the rate constants of activation and deactivation, thereby shifting the ATRP equilibrium. This, in turn, regulates catalyst activity and the polymer bonds’ stability. Among various solvent characteristics, the dipolarity/polarizability parameter, which reflects nonspecific interactions between solutes and solvents, has the greatest effect on the activation rate constant.^67^ An increase in solvent polarity results in a faster activation step and a slower deactivation step, which ultimately leads to a higher *K*_ATRP_ equilibrium constant. Such behavior has been observed in polar organic solvents, such as dimethyl sulfoxide (DMSO). Moreover, the formation of hydrogen bonds and Lewis acid–base complexes involving the copper catalyst can significantly influence the polymerization process in certain solvents. Solvation effects are more pronounced for the deactivator complex (Cu^II^/L) than for the activator (Cu^I^/L), due to the higher polarity of the former and solvent-dependent halidophilicity, i.e., X–Cu^II^/L stability.^68–72^ This difference in solvation between the two copper oxidation states largely explains the solvent-dependent variations in the activation and deactivation rate constants, as well as the overall ATRP equilibrium.^62,73^

In RAFT polymerization, the effect of the solvent primarily depends on the type of monomers and chain transfer agents (CTAs) used. For monomers capable of forming intermolecular hydrogen bonds, aprotic solvents are generally unfavorable, as they do not disrupt the formation of hydrogen bonds between polymer chains. This can lead to polymerization retardation, low monomer conversion, and broad molecular weight distribution. To achieve well-controlled polymerization, it is therefore recommended to use water or a mixture of protic and organic solvents. The variety of available CTAs presents a major challenge in selecting a suitable solvent for polymerization. All reactants must be solubilized throughout the polymerization process, unless working in dispersed media. Additionally, the selected solvent must not negatively impact the activity and stability of the CTA, as this could result in significant deviations from the theoretical molecular weight.^63,64^

To date, organic solvents such as anisole, toluene, acetonitrile, methanol, ethanol, 1,4-dioxane, *N*,*N*-dimethylformamide (DMF), formamide, dimethyl sulfoxide (DMSO), dimethylacetamide (DMAc), or tetrahydrofuran (THF) have been widely used as reaction environments for controlled polymerization. These solvents offer excellent control over polymerization, resulting in polymers with narrow molecular weight distribution.^62–64,73,74^ Beyond their established roles, some organic solvents can serve as a radical source in ultrasound-mediated controlled polymerization, thereby eliminating the need for conventional radical initiators.^75^ Furthermore, polar solvents have been shown to enhance the activation rate constant and preferentially stabilize the Cu^II^ complex over Cu^I^, improving control in ATRP systems.^73^ Despite these advantages, many organic solvents are toxic, volatile, and pose a significant risk to both human health and the environment. Consequently, their use is also subject to restrictions under the Registration, Evaluation, Authorization, and Restriction of Chemicals (REACH) regulation, leading to a notable decline in their industrial applications. Particularly concerning is the widespread use of DMF, which has been identified as a reproductive health hazard. As a result, the European Union has imposed restrictions on its use in academic and industrial areas from December 2023, and has strongly recommended the substitution of DMF with safer alternatives.^59,76^ Although these conventional solvents are inexpensive and readily available on an industrial scale, they are usually used in large quantities, contributing significantly to chemical waste and raising environmental and safety concerns.^2^

Therefore, the ecological alternative in this case is an aqueous environment. But its widespread application is primarily limited by the solubility of all components within the reaction mixture across various RDRP techniques. A key concern in RAFT polymerization is the possible hydrolysis of chain transfer agent during the polymerization, especially thiocarbonylthio species, as well as the hydrolysis of monomer or polymer. The resulting hydrolysis products can adversely react with CTA and reduce the availability of thiocarbonylthio end groups on the polymer chain, leading to undesired termination events that are difficult to control.^77,78^ These hydrolysis reactions are accelerated at higher temperatures and elevated pH levels. In addition to hydrolysis, aminolysis by primary and secondary amines – either present in the reaction mixture or formed as a result of monomer hydrolysis. However, this destructive effect can be significantly mitigated by lowering the pH of the reaction media, thereby protonating the amine-containing compounds and reducing their nucleophilicity.^64,79^

The use of aqueous ATRP introduces additional challenges, such as the catalytic complex, propagating radicals, and initiators are susceptible to various side reactions listed in Figure 4. In water, both the ATRP equilibrium constant (*K*_ATRP_ = *k*_act_/*k*_deact_) and the activation rate constant (*k*_act_) are high. Moreover, both the activator and deactivator complexes are sensitive to disproportionation and halide dissociation, respectively. Generally, disproportionation is more favorable in more polar solvents. However, solvents that coordinate with Cu^I,^ such as DMSO or MeCN, can effectively suppress this process.^80–82^ Disproportionation of activator can also be limited by the proper choice of ligand, characterized by high Cu^I^/L and Cu^II^/L stability constants, e.g., tris­(2-pyridylmethyl)­amine (TPMA), and by tuning the concentration of free ligand.^62^ Commonly, ATRP systems use at least a 2-fold excess of free ligand.^83^ A significant fraction of the deactivator (X-Cu^II^/L) dissociates into halide ions and inactive Cu^II^/L species, which lowers the concentration of deactivator. This leads to a high concentration of radicals, increasing the rate of chain termination and contributing to the formation of dead polymer chains. In addition, high polarity of water can cause hydrolysis of ATRP initiator and dormant polymer chains. Water also promotes the solvation of halide ions in the deactivator complex via hydrogen bonds, further reducing their concentration. This shifts the equilibrium of the reaction toward the formation of active radicals, since the Cu^II^ complex becomes less effective in deactivation. These findings support previous observations that ATRP in aqueous and protonic environments tends to proceed at faster polymerization rates, but with diminished control over polymer chain length and molecular weight distributions.^62,84,85^

**Figure 4:**
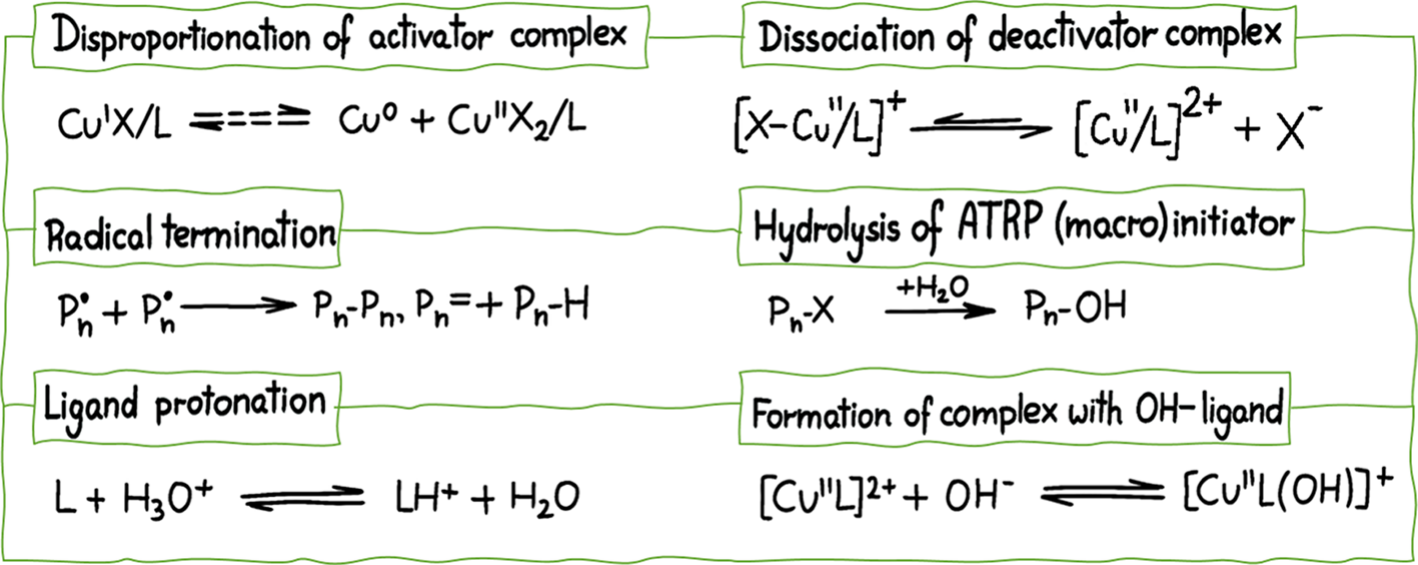
Potential side reactions during ATRP in an aqueous medium.^70,85^

Despite the aforementioned limitations, ATRP can be successfully conducted in aqueous media by carefully adjusting the reaction conditions to favor the equilibrium toward the deactivator, while maintaining a sufficient concentration of the activator in the reaction mixture.^70^ These optimized conditions comprise: (1) achieving a high molar ratio of Cu^II^ to Cu^I^ by providing a suitable Cu^I^ regeneration system; (2) adding excess halide salts to suppress halide ion solvation; (3) using ligands that form stable complexes with copper, along with an excess of ligand to enhance complex stability; (4) adjusting the pH to prevent protonation of the ligands at low pH, or stronger complexation with hydroxide ions at high pH. These challenges make controlling ATRP in water much more demanding than in organic solvents, where activation rates are considerably slower but more predictable. Noteworthy results have been achieved while employing untreated water (river, stream, rain, spring, and sea) without any purification.^86^ An alternative approach involves using a mixture of organic solvents and water, which enables faster polymerization while maintaining the desired control over the process.^87^ From both environmental and economic standpoints, ethanol/water mixture, especially when derived from commercially available alcoholic beverages, has shown promising results in the photo-ATRP of 2-(dimethylamino)­ethyl methacrylate (DMAEMA).^88^ Interestingly, the presence of sugars or aromatic compounds such as coumarin derivatives in flavored alcoholic beverages significantly accelerated poly­(2-(dimethylamino)­ethyl methacrylate) (PDMAEMA) growth compared to other “pure” alcohols, due to enhanced catalyst regeneration. Polymerization rates in these beverages were observed to be more than 20 times faster than in distilled water. These beverages have gained interest as nonconventional solvents for RDRP due to their multifunctional role: they can serve as reducing agents, supplemental activators, and cost-effective solvent alternatives.^24^ Alcoholic beverages such as flavored beers, wines, brandies, liqueurs, ciders, and cocktails are particularly attractive because they contain relatively high concentrations of antioxidants, including l-ascorbic acid and citric acid, as well as reducing sugars like fructose and maltose. These components contribute to increased polymerization rates by facilitating the regeneration of the active catalyst. Another notable class of nonconventional solvents for RDRP includes biological media, such as fetal bovine serum, calf serum, fermentation broth, lysogeny broth, and phosphate-buffered saline. These biocompatible environments enable the synthesis of advanced bioconjugates, such as polymer–DNA hybrids,^89^ and protein–polymer hybrids.^90–92^

Nevertheless, the use of water as a solvent in RDRP is limited by the hydrophobic nature of many monomers and polymers, as well as the moisture sensitivity of various reagents and catalysts. To address these challenges and to avoid the use of toxic, expensive organic solvents, polymerization in heterogeneous media was employed. Depending on the size of the dispersed droplets, the heterogeneous systems can be classified as follows: suspension (<2 mm),^93^ emulsion (>100 μm),^94^ miniemulsion (<500 nm),^20,95^ or microemulsion (<50 nm).^96,97^ Dispersion systems offer several advantages. They enable effective heat transfer, with the continuous phase acting as a heat-dissipating element for exothermic processes occurring in micelles suspended in the continuous phase. Moreover, unlike bulk and solution polymerization, these types of systems allow for reduced viscosity of the reaction mixture, predominantly influenced by the continuous phase, thereby facilitating easier handling and processing.^96,98^ Aqueous dispersion media, in particular, offer distinct advantages over bulk or solution media. Under optimized conditions, polymerization in dispersion can enhance control over the reaction, enable the synthesis of high molecular weight polymers, and remarkably improve the performance of complex polymer architectures and hybrid nanoparticles.^32,96,99,100^ Moreover, the biphasic nature of heterogeneous systems can facilitate the removal of catalysts. For instance, in emulsion polymerizations, the large interfacial area between the aqueous and organic phases promotes the migration of the catalyst from polymer particles into the aqueous phase, provided the catalyst possesses adequate hydrophilicity. At the same time, the catalyst should possess sufficient hydrophobicity to penetrate the hydrophobic monomer droplets, where emulsion polymerization is initiated and controlled.^96,101^ To achieve stable dispersion, it is necessary to use surfactants (either ionic^22,102^ or nonionic^103^) along with cosurfactants. These aid in stabilizing micelles formed through mechanical agitation or sonication. However, selecting a suitable surfactant and cosurfactant system can be particularly challenging in ATRP, as anionic surfactants may interact with the catalytic complex, negatively affecting its reactivity and stability. The main drawback of using surfactants is the potential contamination of the final polymers, the generation of additional waste products, and thus the necessity for purification procedures. Residual surfactant can impair the electric, photonic, and surface properties of the products.^96,99^ To overcome these limitations, soap-free emulsion polymerization was developed.^104^ This approach involves the use of a “reactive surfactant”, which serves multiple functions in the reaction mixture. These compounds can simultaneously act as surfactants and as monomers, ligands, chain transfer agents, or initiators, offering a multifunctional solution to improve reaction efficiency while reducing impurities.^105^

In traditional dispersion systems, the continuous phase typically consists of water combined with a surfactant, which serves as the primary dispersing medium for insoluble or low-soluble monomers. Along with the initiator and cosurfactant, these monomers create the dispersed phase, eliminating the need for using toxic organic solvents.^96^ An alternative and noteworthy approach is the inverse emulsion system, where the organic phase constitutes a continuous phase while the aqueous phase is dispersed. Both inverse and conventional emulsion RDRP offer numerous processing advantages, including polymerization within monomer droplets and the elimination of monomer and catalyst transport limitations. However, to date, the synthesis of hydrophilic polymers via inverse emulsion has primarily used systems wherein the continuous phase is composed of hexane or cyclohexane, both of which are toxic.^106^ Therefore, replacing such harmful solvents is a critical step toward aligning the development of polymerization methods with green chemistry principles. Recent studies have highlighted a successful methodology for employing vegetable oils as a continuous phase within inverse emulsion systems, marking a significant advancement in sustainable polymerization practices.^107,108^ These examples are further discussed in Section 3.3.

Solubility discrepancies can also be strategically exploited in emulsion polymerization and polymerization-induced self-assembly (PISA) processes. In these systems, solvent selectivity toward one polymer block enables the emulsion polymerization of a second block. Consequently, amphiphilic block copolymers spontaneously self-assemble in a one-step process, as the growth of the insoluble block drives the formation of well-defined structures such as micelles, lamellae, or worm-like morphologies. Despite the advantages offered by these alternative strategies, they often present additional challenges. They may include a limited monomer scope, particularly in aqueous dispersion, or the necessity for multiple purification steps, depending on the system.^109–111^

In parallel, extensive efforts have been made to explore green solvents for the RDRP of various monomers.^8^ Among these, ionic liquids (ILs) have garnered significant interest due to their unique properties, such as low volatility, high thermal stability, and tunable solvating abilities.^112–115^ However, most of them are nonbiodegradable and hazardous, and require complex synthesis procedures. Their behavior in a polymerization system depends heavily on the specific combination of ions used, which can lead to properties that are volatile, flammable, or otherwise hazardous, potentially undermining their viability as truly green alternatives.^116^

Given the limitations of ILs, deep eutectic solvents (DESs), also known as eutectic mixtures (EMs), have also been explored as a promising alternative.^116,117^ EMs are composed of a hydrogen bond acceptor (HBA) and a hydrogen bond donor (HBD), which, at a specific temperature and molar ratio, form a mixture with a melting point lower than that of the individual components.^118^ EMs have recently gained the attention of the scientific community due to several favorable properties, such as low cost, low toxicity, low volatility, reduced flammability, and ease of preparation.^118–121^ However, certain drawbacks remain, such as high viscosity and the tendency to solidify at room temperature. While studies suggest that most DESs do not cause environmental pollution upon release,^116^ the questions of toxicity and biodegradability remain unresolved and require further investigation. The toxicity and cytotoxicity of DESs are dependent on the structures of HBA and HBD used, making it challenging to draw definitive conclusions.^122^ A significant advancement in addressing these concerns lies in the ability to synthesize EMs from naturally sourced materials, which improves their sustainability and biocompatibility.^123–125^ Given these facts, the use of EMs in polymer synthesis has been thoroughly reviewed.^114,126,127^ Several recent studies on biomass-derived EMs are discussed in Section 3.2.

Another environmentally friendly solvent used in RDRP is supercritical carbon dioxide (CO_2_). It is nontoxic, cost-effective, and easily recyclable.^128,129^ However, the main obstacle to its widespread implementation is the selective solubility of the reaction mixture components used in the RDRP.^15,130^

Another environmentally friendly solvent used in RDRP is supercritical carbon dioxide (CO_2_). It is nontoxic, cost-effective, and easily recyclable.^128,129^ However, the main obstacle to its widespread implementation is the selective solubility of the reaction mixture components used in the RDRP.^15,130^

In the pursuit of more sustainable methods, new biomass-derived solvents have been applied in RDRP techniques such as ATRP and RAFT to synthesize well-defined macromolecules with tailored functionalities. Thus, far, solvents derived from (1) lignocellulosic biomass, (2) essential oils, and (3) vegetable oils have been successively applied in controlled polymerization processes (Figure 5). Current research focuses on replacing toxic, petroleum-derived ethereal and dipolar aprotic solvents with renewable alternatives, guided by similarities in their HSP and KAT parameters.

**Figure 5:**
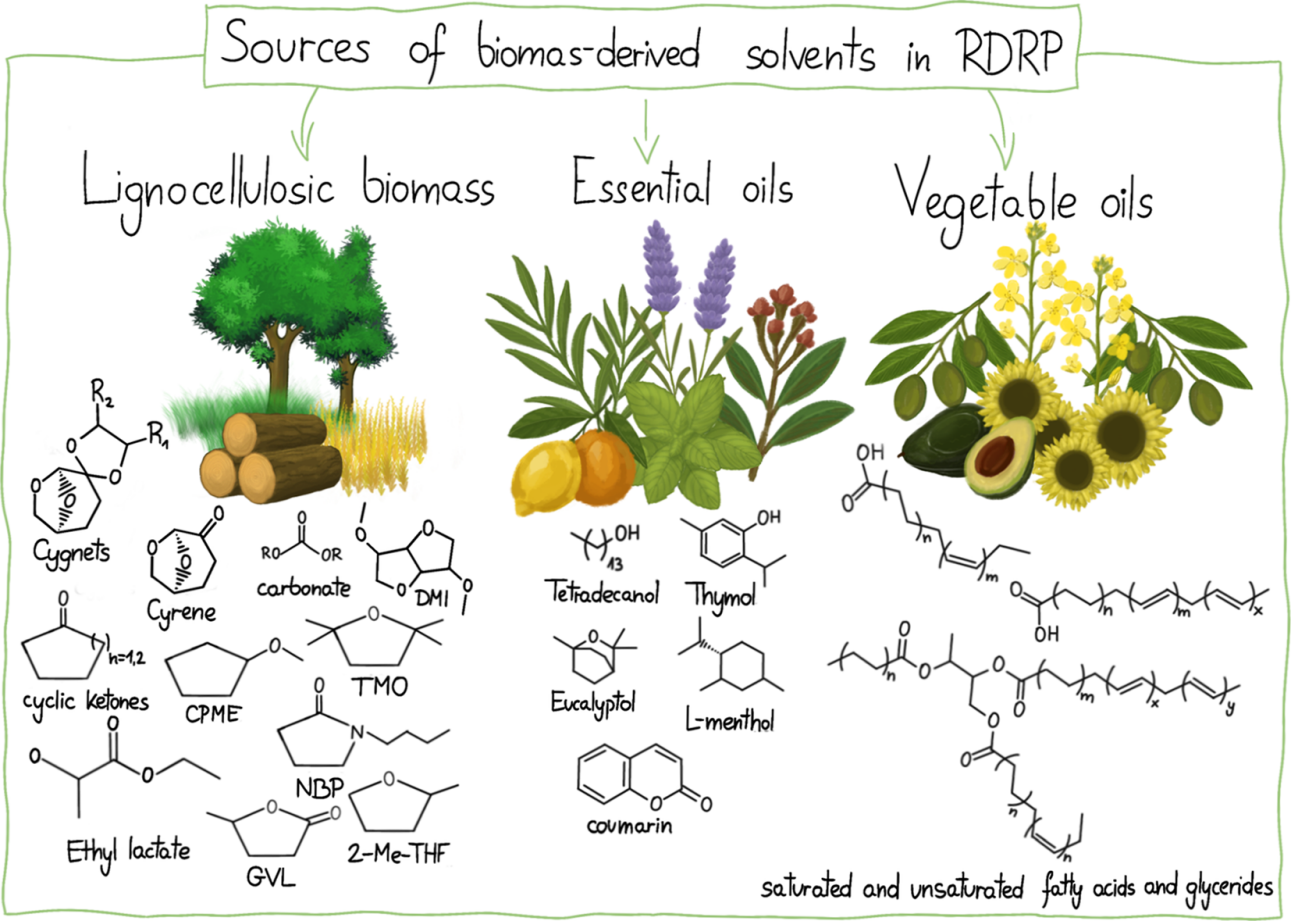
Examples of biomass-derived solvents used in RDRP.

### 3.1 Lignocellulosic Biomass-Derived Solvents

Until now, research has primarily focused on lignocellulose-derived decomposition products such as Cyrene, Cygnet, γ-valerolactone (GVL), 2-methyltetrahydrofuran (2-Me-THF), cyclopentyl methyl ether (CPME), ethyl acetate (EtOAc), and ethyl lactate (EL). The following subsection presents a detailed analysis of the highly promising results obtained using these renewable solvents in RDRP techniques.

Cyrene, from a structural point of view, belongs to the class of bicyclic ketones. Given its polarity, defined by both KAT parameters and HSP, it serves as a potential substitute for common toxic solvents such as dichloromethane (DCM), *N*-methylpyrrolidone (NMP), DMSO, DMF, and DMAc. Cyrene is synthesized in two steps from cellulosic biomass^131–133^ (see Figure 6). Notably, it is nonmutagenic, with a median lethal dose (LD_50_) greater than 2000 mg/kg, which is significantly higher than the threshold of toxicity of solvents (50 mg/kg) (see Table 1).

**Figure 6:**
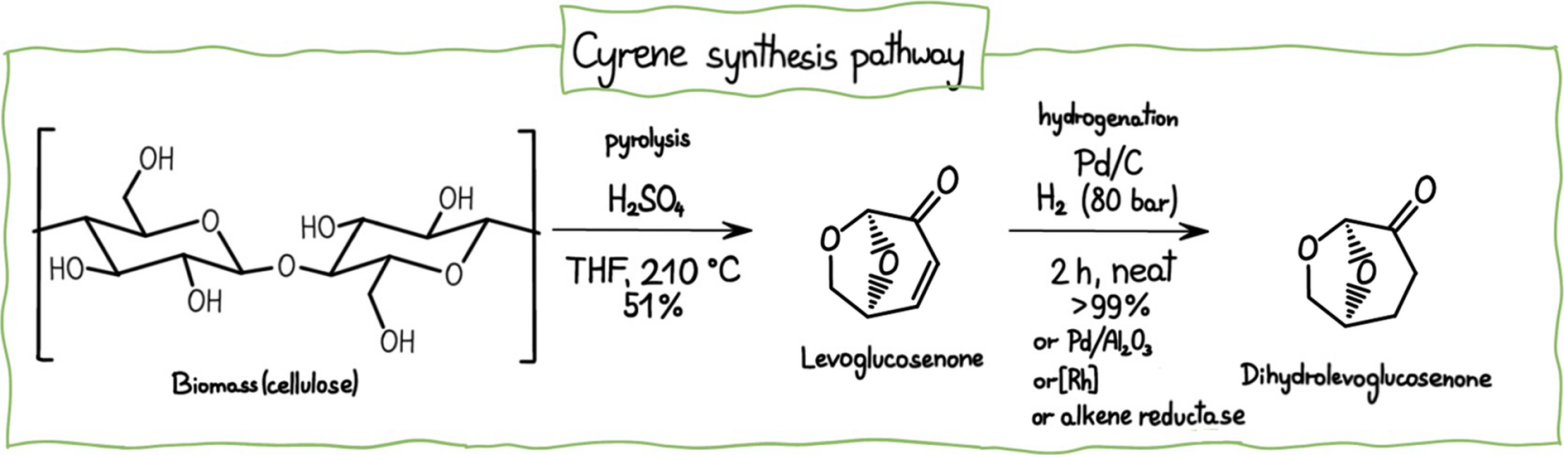
Cyrene synthesis pathway. Adapted with permission under a Creative Commons CC-BY 4.0 from ref 2. Copyright 2022 American Chemical Society.

Research has demonstrated that Cyrene can effectively function as a substrate for the synthesis of renewable monomers^134^ and various solvents, including ketals. These ketals, referred to as Cygnet-type compounds (e.g., Cygnet 0.0), are obtained via catalytic reaction of dihydrolevoglucosenone with ethylene glycol in the presence of an acid catalyst, forming a dioxolane ring (Figure 7).^135^ Cygnet 0.0 is considered a possible replacement for DCM.^57,135,136^

**Figure 7:**
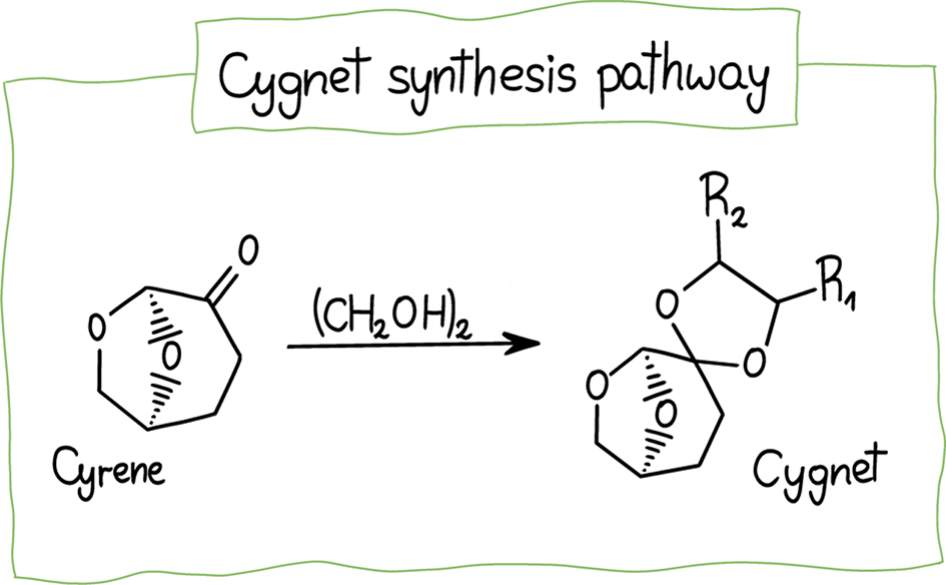
Cygnet synthesis pathway. Adapted with permission from ref 135. Copyright 2016 Wiley-VCH Verlag GmbH & Co. KGaA, Weinheim.

The use of Cyrene as a solvent in polymer synthesis remains relatively underexplored, but promising examples have emerged. It has been successfully applied in the synthesis of polymers via ATRP^137–139^ and RAFT,^140^ demonstrating its potential as a sustainable replacement for toxic dipolar aprotic solvents.

Well-defined polymers with narrow MWD, high conversions, and high chain-end fidelity have been successfully synthesized in Cyrene, even without applying external deoxygenation or additives.^137^ A wide range of hydrophobic monomers, including methyl acrylate (MA), methyl methacrylate (MMA), *tert*-butyl acrylate (*t*BA), benzyl acrylate (BzA), trifluoroethyl acrylate (TFEA), and styrene (St), have been successfully polymerized. Initial experiments involved MA polymerization under an inert nitrogen atmosphere using Cu(0) wire, 1000 ppm of CuBr_2,_ and tris­[2-(dimethylamino)­ethyl]­amine (Me_6_TREN) as catalytic complex. Conducted at ambient temperature, the polymerization yielded a high monomer conversion of 90%, MWD of 1.08, and molecular weight (MW) of 4,800, with an initiation efficiency approaching 100% over 18 h. Similar results were obtained for MMA under analogous conditions, yielding almost quantitative conversion (97%), MWD of 1.11, and MW of 7,200, with satisfactory initiation efficiency of 88%. Encouraged by these results, the authors explored simplifying the protocol by conducting polymerizations under fully oxygenated conditions, but this required only 3 h. For other monomers, polymerization times ranged from 20 to 45 h, still yielding high conversions (75–94%) and low dispersities (MWD = 1.11–1.37). These outcomes were comparable to Cu(0)-RDRP without external deoxygenation in commonly used toxic organic solvents.^141^ Additionally, controlled polymerization of MA was achieved at extremely low CuBr_2_ concentrations, as low as 15 ppm. Under nondeoxygenated conditions, poly­(methyl acrylate) (PMA) with molecular weights ranging from 700 to 28,000 and narrow MWD was obtained by targeting degree of polymerization (DP) from 5 to 800. For lower DP, theoretical and experimental MW values aligned well within 3 h. However, higher DP showed reduced initiation efficiency and required longer polymerization times (24–96 h). To further confirm the fidelity of chain ends, the authors synthesized AB and ABA block copolymers (A = MA, B = EA) via sequential monomer addition, resulting in a precisely defined triblock copolymer, even under oxygenated conditions. Despite some challenges in synthesizing polymers with very high molar masses or the formation of a biphasic system during *t*BA polymerization, Cyrene presents substantial benefits in sustainable polymer chemistry.

Recently, Cyrene was successfully used as a solvent for Cu(0)-mediated RDRP of terpenoid-based geranyl acrylate.^139^ The monomer, by its nature, can be demanding due to the presence of two double bonds in the side chain. These may lead to potential challenges during polymerization, such as branching, cross-linking, but also offer opportunities for postpolymerization modification. The authors investigated two ATRP methods, using Cu(0) wire- and light with Cu­(II)/L as deactivator.^81^ The SARA ATRP with Cu(0) wire was conducted with 5 cm of preactivated copper wire, ethyl α-bromoisobutyrate (EBiB) as initiator, and relatively high catalyst loading ranging from 1,000 to 3,000 ppm in the form of Cu^II^Br_2_/Me_6_TREN. Higher catalyst loading yielded higher conversion, however, an increase in dispersity was observed. The best results were achieved at room temperature. Notably, SARA ATRP in Cyrene delivered higher conversions and relatively low dispersities at room temperature, even without trifluoroethanol as a cosolvent. Conversely, photoinduced ATRP in Cyrene yielded lower conversion, molecular weight, and higher dispersity compared to SARA ATRP. The studies^137,139^ have proven Cyrene as a successful biobased replacement to conventional organic solvents for RDRP employing Cu(0) wire, particularly in the synthesis of hydrophobic polymers. These findings reinforce its suitability for sustainable polymerization processes.

Additionally, the insights gained from polymerizing both hydrophilic and hydrophobic monomers in ATRP using Cyrene have significantly broadened its potential applications. The utility of a new Cyrene-derived solvent, Cygnet 0.0, has also been explored and shows promising results (Figure 8).^138^

**Figure 8:**
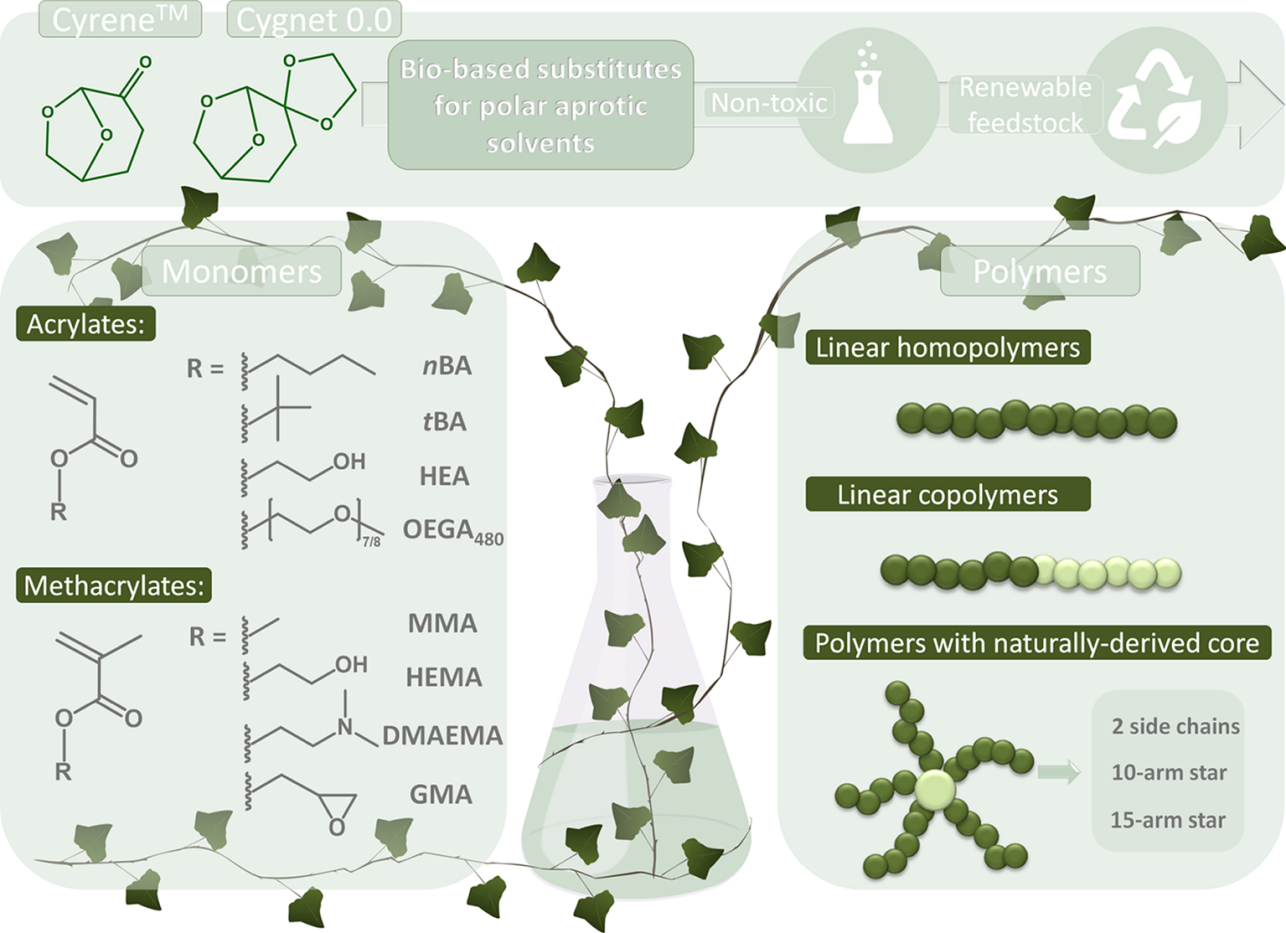
Range of applicability of biomass-derived solvents in polymerization of (meth)­acrylates via ATRP techniques. Adapted with permission under a Creative Commons CC-BY 4.0 from ref 138. Copyright 2024 The Authors. Published by American Chemical Society.

Cyrene and Cygnet 0.0 have been successfully applied as solvents for ATRP of *n*-butyl acrylate (*n*BA) as model monomer, polymerizing it from small molecular weight initiators, as well as macroinitiators derived from riboflavin (vitamin B_2_), troxerutin, and β-cyclodextrin. The first attempt was to compare the same reaction conditions using two different solvents: Cyrene or DMF. The reaction employed EBiB as the initiator, Cu^II^Br_2_/TPMA (300 ppm) as the catalytic complex, and copper wire as a supplemental activator and reducing agent in SARA ATRP. While the polymerization of *n*BA in DMF was well-controlled and completed in just 2.5 h, the same process in Cyrene required approximately 100 h to achieve similar results. This prompted multiple attempts to optimize the polymerization conditions in Cyrene. Adding multiple portions of copper wire during the polymerization reduced the reaction time but led to a high initiation efficiency (*I*_eff_) of approximately 200%, indicating the presence of chain activity transfer during polymer growth. Switching the reducing agent to ascorbic acid in ARGET ATRP resulted in only oligomer formation. This outcome was linked to the potential reaction of the hydroxyl groups of ascorbic acid with a ketone group of Cyrene, forming hemiketals or ketals in the presence of a strong acid such as HBr, formed during the reduction of Cu^II^Br_2_ with ascorbic acid.^142^ Importantly, cyclic voltammetry measurements of the Cu^II^Br_2_/TPMA in both DMF and Cyrene revealed a significantly reduced cathodic current and an absence of anodic peaks in Cyrene, suggesting that Cyrene reacts with the catalytic system. This interaction likely reduces the effective catalyst concentration in the reaction mixture compared to DMF under otherwise identical conditions.^143^ To address this, alternative ligands were explored to stabilize the catalytic complex. Me_6_TREN emerged as the most effective ligand, resulting in a nearly 40-fold increase in polymerization rate, comparable to Cu^II^Br_2_/TPMA in DMF. Further experiments demonstrated that Me_6_TREN facilitated well-controlled polymerization of various (meth)­acrylates, including *t*BA, 2-hydroxyethyl acrylate (HEA), MMA, 2-hydroxyethyl methacrylate (HEMA), poly­(ethylene glycol) methyl ether acrylate (OEGA_480_), glycidyl methacrylate (GMA), and DMAEMA, with a relatively narrow MWD of 1.15–1.50 and high conversions up to 89%. However, some challenges remained. MMA, also achieving high conversion, showed limited molecular weight development (*M*_W_ ≈ 1700), likely due to excessive radical generation. Polymerization of OEGA_480_ required extended reaction times, while HEA only reached 22% conversion. The authors also verified chain-end functionality by performing a chain-extension experiment with a second poly­(*n*-butyl acrylate) (P*n*BA) block, resulting in polymers with a narrow molecular weight distribution (MWD = 1.11). Based on optimized conditions for P*n*BA synthesis in Cyrene, Cygnet 0.0 was also evaluated. Due to its physicochemical properties, the syntheses were carried out at 75 °C, producing polymers with narrow MWD values of 1.12–1.21 and closer agreement between experimental and theoretical molecular weight compared to reactions performed under analogous conditions in Cyrene. Notably, the Cu^II^Br_2_/Me_6_TREN catalytic complex exhibited reversible redox behavior in Cygnet 0.0, indicating the absence of side reactions. This allowed decreasing the catalyst concentration even to 75 ppm, still maintaining the controlled character of *n*BA polymerization. In contrast, reducing the catalyst concentration below 100 ppm in Cyrene caused a significant slowdown in the reaction. As with Cyrene, a variety of monomers were successfully polymerized in Cygnet 0.0. Remarkably, HEA polymerized exhibited 17.5 times faster than *n*BA under the same catalytic concentration, reaching 92% monomer conversion in 14 min, with a relatively low dispersity (MWD = 1.36), though the molecular weight was overestimated – a known phenomenon for HEA due to its interaction with the chromatography column.^144^ For the methacrylates – HEMA and MMA, performance was suboptimal, with low conversions, long reaction times (up to 25 h), and significant deviations in molecular weight.

Moreover, the feasibility of synthesizing branched systems by functionalizing riboflavin, troxerutin, and β-cyclodextrin has also been confirmed. These yielded polymers with 2, 10, or 15 P*n*BA side arms, and narrow MWD: for Cyrene^TM,^ ranging from 1.24 to 1.54, and for Cygnet 0.0, ranging from 1.31 to 1.41.^138^ Overall, this comprehensive study presents a compelling case for replacing toxic polar aprotic solvents with Cyrene and Cygnet 0.0. The polymerization rates and quality of the formed polymers are comparable, supporting their adoption in green and sustainable chemistry.

In addition to its successful application in ATRP techniques, Cyrene has also shown promise as a solvent in RAFT polymerization, further highlighting its versatility in polymer chemistry.^140^ This research represents the pioneering application of Cyrene as a solvent in the RAFT polymerization of the biobased lactone monomer γ-methyl-α-methylene-γ-butyrolactone (γMeMBL). Overall, RAFT polymerization in both Cyrene and DMSO demonstrated good control over molecular weights, low dispersity, and first-order kinetics, with a linear increase of molecular weight as monomer conversion proceeded. Polymerizations conducted in Cyrene generally resulted in slightly lower conversions compared to those in DMSO under identical conditions. At a lower degree of polymerization (DP = 50), Cyrene yielded a better agreement between theoretical and experimental molecular weight, along with a narrower MWD (MWD = 1.25) compared to DMSO (MWD = 1.31 for DMSO). As the targeted DP increased, both solvents showed a decrease in conversion and dispersity, likely due to a lower initiator concentration. Minor deviations in molecular weight were also observed at higher DPs in both solvents. The synthesized P­(γMeMBL) homopolymers exhibited high glass transition temperatures (206–221 °C), dependent on molecular weight, and excellent thermal stability, with the onset of degradation in the range of 345–366 °C. These properties are significantly better than those of poly­(methyl methacrylate) (PMMA). Recent studies affirm that Cyrene is an efficient and sustainable alternative to traditional toxic solvents in the synthesis of high-performance polymers. With successful outcomes in the polymerization of both hydrophilic and hydrophobic monomers, Cyrene not only meets the performance demands of modern polymer synthesis but also aligns with sustainable practices, making it a transformative solvent in the field.

Lignocellulosic biomass serves as an excellent source for producing a wide array of chemicals, notably including a variety of solvents such as the aforementioned Cyrene as well as GVL and its derivatives, namely γ-butyrolactone, γ-caprolactone, and γ-octanolactone. GVL is synthesized via the hydrogenation of levulinic acid (LAc), a platform chemical derived from the acidic conversion of cellulose and hemicelluloses^145^ (Figure 9).

**Figure 9:**
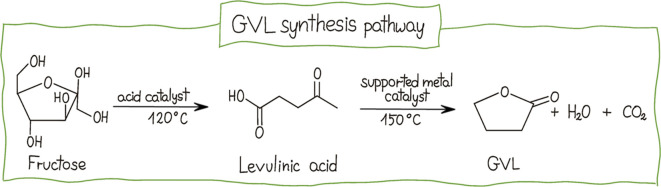
GVL synthesis pathway. Adapted with permission from ref 145. Copyright 2021 Royal Society of Chemistry.

Considering its solubility parameters (see details in Table 1), GVL can serve as a viable alternative to DMF, DMAc, and NMP. It is characterized by low toxicity (LD_50_ oral rats = 8,800 mg/kg) and low volatility, which reduces flammability under standard conditions.^52,145,146^ The successful examination of GVL as a solvent for ATRP of methacrylates and styrene was recently carried out and discussed in the following research publications.^147,148^

One study explored Fe­(III)-catalyzed AGET ATRP in GVL of various monomers, including MMA, ethyl methacrylate (EMA), butyl methacrylate (BMA), and benzyl methacrylate (BzMA).^147^ The primary focus was the polymerization of MMA (DP = 200) with 2500 ppm FeBr_3_ as the catalyst, ethyl α-bromophenylacetate (EBPA) as the initiator, and sodium ascorbate (AsAc-Na) as the reducing agent. The results showed that GVL could act as both a solvent and a ligand, coordinating with iron halides through the −OCO– functional group. The applied system resulted in 70% monomer conversion, a narrow MWD of 1.27, and initiation efficiency around 90%, which indicates good control over molecular weight. Notably, effective polymerization control was maintained even with FeBr_3_ concentrations as low as 50 ppm, though further reduction to 5 ppm resulted in loss of control. Increasing the temperature accelerated polymerization, but also increased termination events, with 75 °C identified as the optimal temperature. Further AGET ATRP was conducted using FeBr_3_/GVL as a catalytic system, polymerizing a series of methacrylates: EMA, BMA, BzMA, to further explore the system’s versatility and potential applications. All tested methacrylates exhibited good agreement between theoretical and experimental molecular weight, and moderate MWD ranging from 1.24 to 1.32. Slightly lower conversions were obtained (50–60%) compared to MMA. In an extension of this approach, the authors applied the nontoxic iron catalyst system to other nonpolymerizable GVL derivatives. Therefore, the polymerization of MMA was conducted in γ-butyrolactone, γ-caprolactone, and γ-octanolactone, yielding similar results, supporting the hypothesis that the nonpolymerizable five-membered lactone ring contributes to the solvent’s efficiency, unlike ε-caprolactone, which can polymerize. Moreover, the livingness of the resulting polymers was confirmed through a chain-extension experiment under the same conditions, further validating the control offered by this green polymerization system.^147^

In addition to methacrylates, GVL is an efficient solvent for styrene polymerization.^148^ It is important to know that styrene presents a significant challenge in ATRP. It is caused by the potential of β-H elimination, catalyzed by the Cu­(II) deactivator complex, and bimolecular coupling.^149–151^ Consequently, this leads to a loss of chain-end functionality and a cessation of polymerization. Initial attempts at polymerizing styrene in GVL applied a CuBr_2_/TPMA/AsAc-Na catalyst system, and yielded polymers with a moderately successful conversion. However, these polymers exhibited poor control over dispersity, resulting in MWD exceeding 1.30. The addition of Na_2_CO_3_ yielded superior results, achieving high yields and narrow MWD below 1.3, with very low amounts of used catalyst (∼5 ppm). NMR spectroscopy, chain extension analysis, and matrix-assisted laser desorption/ionization mass spectrometry (MALDI-MS) experiments collectively confirmed the high fidelity of the synthesized polymer chains, thereby establishing the GVL as a suitable and safer replacement for toxic solvents, even in the more demanding polymerization reactions involving challenging monomers with very low catalyst consumption.^148^ Moreover, the study^147^ has demonstrated that GVL can also serve as a ligand in Fe-catalyzed AGET ATRP of methyl methacrylate, thereby eliminating the need for any additional ligands and offering a key benefit.

Recent studies have extensively investigated the polymerization of styrene using a mixture of biomass-derived solvents, specifically EtOAc and ethanol (EtOH).^152–157^ While ethyl acetate is still predominantly produced from fossil resources, it can be sustainably synthesized through microbial fermentation of sugar-rich waste from the food industry.^158,159^ Ethanol, a well-known biomass-derived solvent, is typically produced via fermentation of carbohydrate-rich feedstocks.^160^ It is a common protic green solvent, likely used as a cosolvent, accelerating the polymerization rate. Due to its broad investigation in RDRP techniques,^24^ ethanol is considered beyond the primary scope of this review. Blending renewable solvents to replace toxic ones not only reduces reliance on nonrenewable resources but also helps address waste management challenges, paving the way for a greener and more efficient production method. The EtOAc/EtOH mixture can be considered as a suitable alternative to DCM, offering comparable efficacy and versatility in various applications.^161,162^

This solvent mixture has proven efficient for ARGET ATRP of styrene using various reducing systems composed of ascorbic acid (AsAc) and Na_2_CO_3_ or nitrogen bases. Experiments have demonstrated that ARGET ATRP of styrene can proceed in EtOAc/EtOH using Na_2_CO_3_ alone to regenerate Cu­(I) from Cu­(II).^153^ Moreover, Na_2_CO_3_ not only protects the ligand from protonation but also enhances the activity of the reducing agent, thus accelerating the polymerization rate.^152,155^ Under these conditions, controlled telechelic polystyrene was successfully synthesized. However, at conversions exceeding 50%, control over the process was gradually lost due to the termination reactions. Because Na_2_CO_3_ is insoluble in the solvent mixture, its particle size (granulometry) was also considered as a factor potentially influencing catalyst activity. These findings led the researchers to conduct a series of experiments on monofunctional initiators such as ethyl α-chloro- and α-bromoisobutyrate (ECiB and EBiB, respectively) using the same reducing system, AsAc/Na_2_CO_3,_ with different particle sizes. Excellent control was achieved even with low catalyst loading (60 ppm), surpassing results obtained with a bifunctional initiator and remained effective even in the presence of small amounts of water. The polymerization performance was not significantly affected by changes in Na_2_CO_3_ particle size. Moreover, initiators based on chlorine outperformed their bromine-based counterparts.^155^ Importantly, the study also revealed that under ARGET ATRP conditions with a bifunctional initiator and without cross-linking or branching agents, the solvent mixture plays a key role in the gelation process. Gel formation was found to be strongly influenced by the polarity of the environment, monomer volume, concentrations of AsAc and Na_2_CO_3_, and the ratio of monomer to initiator. Higher ethanol content at lower temperatures promotes gel formation. Conversely, less polar environments and lower AsAc/Na_2_CO_3_ concentrations can facilitate controlled polymerization and slow down or eliminate the cross-linking process.^154,157^

On the contrary, a homogeneous reducing system using isopropylidene ascorbic acid (H_2_AAIPI) with non-nucleophilic nitrogen bases has also been investigated in ATRP of styrene, employing EtOAc/EtOH solvent.^156^ The soluble reducing system identified in this study has proven to be a superior alternative to the previously reported heterogeneous mixture of AsAc and Na_2_CO_3_, particularly when addressing higher molecular weights of the final product. It was observed that nitrogen bases characterized by lower p*K*_a_ values yielded monomodal MWDs with predictable number-average molecular weights at moderate to high conversions. Conversely, when utilizing a base with excessive strength (i.e., higher p*K*_a_), achieving control over the reaction process becomes more challenging.

In addition to the above-mentioned studies on the polymerization of styrene in a mixture of EtOAc/EtOH, one of the first studies in this solvent mixture also demonstrated the ability to achieve controlled conditions in Fe-catalyzed ATRP of acrylamide, obtaining consistent molecular weights and MWD below 1.5.^163^

In summary, extensive research on ethyl acetate mixtures with EtOH has yielded well-controlled processes, thereby confirming the solvent’s versatile potential in the precise synthesis of polymers. The authors assert that this solvent mixture is both more versatile and cost-effective compared to ethyl lactate. However, additional studies involving a variety of monomers are essential to thoroughly investigate the potential of ethyl acetate.

Another notable biomass-derived solvent that has shown promise in the synthesis of versatile macromolecules is 2-methyltetrahydrofuran (2-MeTHF). This solvent offers a safer alternative to carcinogenic DCM and THF, given its solubility parameters and its absence of genotoxicity and mutagenicity.^164^ It is a volatile cyclic ether that can be produced through the chemo-catalytic treatment of furfural or levulinic acid, both derived from renewable lignocellulosic feedstock (Figure 10).^165^

**Figure 10:**
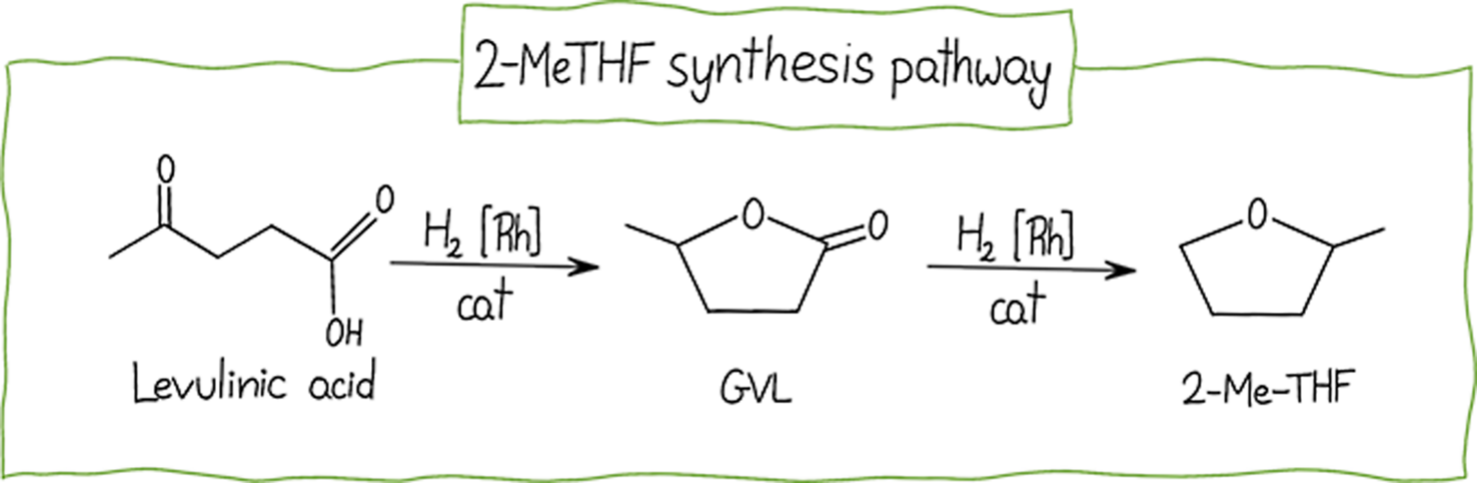
2-MeTHF synthesis pathway. Adapted with permission from ref 166. Copyright 2012 WILEY-VCH Verlag GmbH & Co. KGaA, Weinheim.

Notably, 2-MeTHF can undergo abiotic degradation when exposed to sunlight and air, likely through oxidation and ring opening. These characteristics, along with its renewable origin and environmental degradability, make 2-MeTHF an environmentally attractive solvent. In addition to these benefits, 2-MeTHF has a lower boiling point than some of its analogs (see Table 1 for physicochemical properties), and it can be easily recovered from the solvent through conventional distillation.^166^ A life cycle assessment comparing the production of 2-MeTHF from furfural (obtained from corn-cob waste) with the conventional synthesis of THF demonstrated a substantial environmental advantage for the former, with emissions reduced by up to 97%.^167^

To the best of our knowledge, only two reports have investigated the use of 2-MeTHF as a solvent in RDRP processes, namely RAFT polymerization^168^ and ATRP.^169^ To demonstrate the versatility of 2-MeTHF, various polymerization strategies, including ring-opening polymerization (ROP), free radical polymerization (FRP), and RAFT, were employed both in tandem and separately.^168^ This approach eliminated the need for multiple solvents across different techniques, simplifying the overall synthesis process and reducing solvent consumption. Furthermore, the relatively higher boiling point of 2-MeTHF enables radical polymerizations to be conducted under conditions not feasible with lower boiling point solvents. First, the authors tested ROP for lactide (LA) and caprolactone (CL) using methyl-polyethylene glycol (mPEG) as the initiator. The solvent proved compatible with various catalysts, enabling the synthesis of amphiphilic block copolymers (mPEG–PDLLA, mPEG–PCL) with controlled molecular weights and low dispersities. The feasibility of synthesizing A–B–C block copolymers (mPEG–PCL–PDLLA) in 2-MeTHF was also demonstrated, paving the way for materials with tunable biodegradability.

Furthermore, the applicability of 2-MeTHF was expanded to include ROP-FRP and ROP-RAFT tandem reactions to for the synthesis of amphiphilic hybrid polymers. The solvent demonstrated compatibility with chain transfer agents and azo-initiators, supporting the solubility of all reactants and enabling efficient process execution. As expected, RAFT polymerization showed better control over the process, resulting in amphiphilic biodegradable and nontoxic graft copolymers with a narrow MWD of 1.3 and 1.4 for HEMA and poly­(ethylene glycol) methacrylate (PEGMA) macromonomer, respectively. In contrast, FRP resulted in polymers with broad MWDs, varying from 4.30 (HEMA-LA_25_) to 5.14 (PEGMA-LA_25_). Significantly, the resulting copolymers self-organize into nontoxic nanoparticles, further underscoring the potential of 2-MeTHF as a key tool in the sustainable development of polymeric materials for biomedical applications. The ability to perform multistep polymer syntheses in a single green solvent simplifies production processes and reduces waste generation. The obtained results are auspicious and may provide a valuable source of inspiration for researchers to conduct a more thorough examination of this “multipolymerization” solvent’s potential.

Additionally, 2-MeTHF, along with tetra­(ethylene glycol) dimethyl ether (TEGDME) and CPME (see structure in Figure 5), was successfully employed in photoinduced iron-catalyzed ATRP as well as photoinduced electron-energy transfer (PET) and thermal RAFT.^169^ The study focuses on developing a more environmentally friendly method for polymerizing a range of methacrylate monomers derived from renewable resources such as lignin, including phenyl methacrylate (PheMA), cresol methacrylate (CreMA), guaiacol methacrylate (GuMA), vanillin methacrylate (VaMA), syringol methacrylate (SyrMA)) and terpene (thymol methacrylate (ThyMA), see structure in Figure 11.

**Figure 11:**
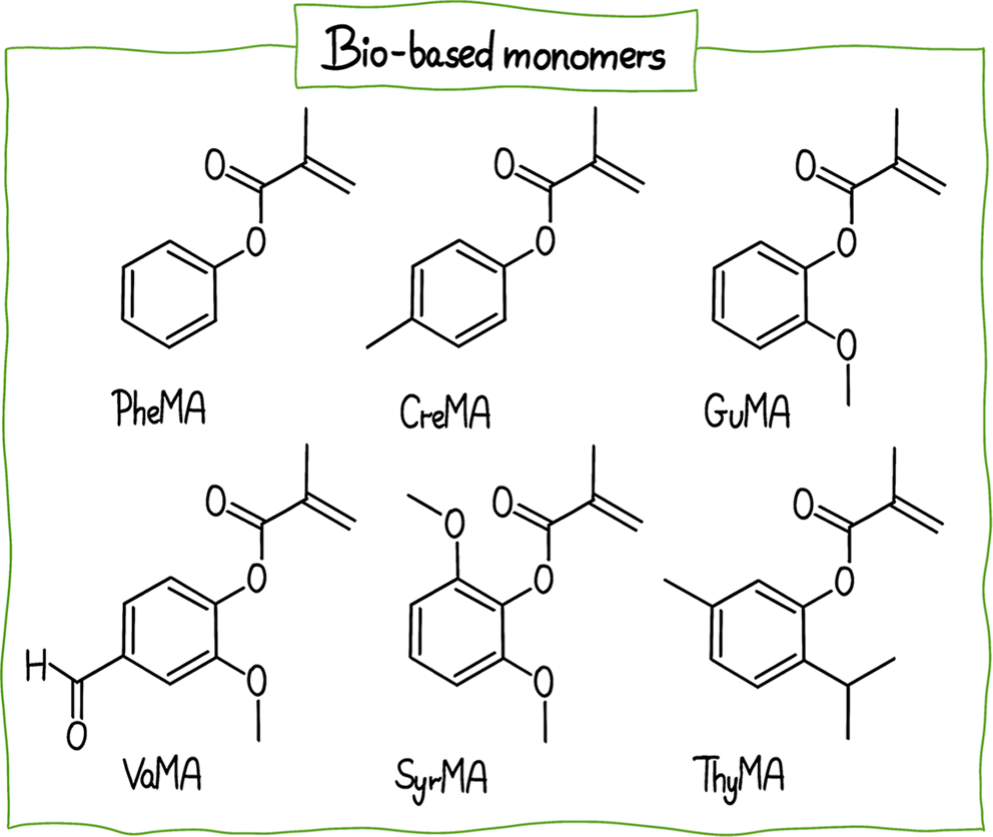
Structure of biomass-derived monomers.

Using 2,000 ppm FeBr_3_/tetrabutylammonium bromide (TBABr) as the catalyst, the polymerizations yielded products with controlled molar masses ranging from 3,400 to 15,100 g/mol and narrow MWDs between 1.16 and 1.28. Notably, the polymerizations could be carried out in the presence of oxygen, simplifying the overall procedure. The results confirmed that photoinduced iron-catalyzed ATRP offers superior control over polymerization, consistently producing narrow MWDs and a close match between theoretical and experimental molar masses across all tested solvents.

The authors pointed out that these biomass-derived solvents did not reduce the activity of the catalyst. In contrast, both PET and thermal RAFT polymerizations exhibited incomplete RAFT agent consumption and higher dispersities ranging from 1.30 to 1.43. Given the increasing emphasis on sustainability, the study underscores the importance of conducting polymer synthesis through environmentally benign methods, particularly by combining biomass-derived solvents, biobased monomers, and biocompatible catalysts to achieve greener polymer production.^169^

The application of the CPME has also been explored in both ATRP^170^ and RAFT polymerizations.^171^ CPME is a hydrophobic ether solvent known for its high boiling point, low toxicity (no evidence of genotoxicity or mutagenicity), and high chemical and thermal stabilities. It is approved for use under the Toxic Substances Control Act (TSCA) and the European List of Notified Chemical Substances (ELINCS). Although CPME can potentially be synthesized from renewable resources, there is currently no commercially available biobased production route. Based on its physicochemical characteristics (see Table 1), CPME is a green alternative to THF and 1,4-dioxane.^2,164,172,173^ CPME was an effective cosolvent, comprising 70% of the mixture alongside water and ethanol in SARA ATRP of a range of monomers, MA, GMA, St, and vinyl chloride (VC). Furthermore, CPME-based mixtures were found to be compatible with a variety of reducing agents, including Fe(0), Cu(0), and Na_2_S_2_O_4_, as well as catalytic complexes, which were not achieved with pure CPME due to limited solubility. When used in the polymerization of MA, the fully green solvent mixture enabled excellent control over molecular weights with targeted DPs in the range of 100–1,000 and MWD ∼ 1.1, yielding high monomer conversion in a relatively short time (up to 8 h), depending on the reducing agent. However, polymerization of other monomers resulted in a broader MWD ranging from 1.17 to 1.58, lower conversions (28% to 70%) when performed in a 70% CPME mixture with either DMF or DMSO. Nevertheless, these findings rival the performance of systems that rely on toxic solvents, highlighting CPME’s potential as a safer alternative.

Later report successfully applied CPME as the sole solvent in RAFT polymerization of MA, St, VC, and vinyl acetate (VAc).^171^ The resulting polymers achieved higher monomer conversion while maintaining the same range of activation rate coefficients as those obtained with THF, DMSO, DCM, and DMF. Additionally, strong agreement was observed between theoretical and experimental molecular weights, indicating well-controlled polymerizations. Notably, the use of CPME enabled a reduction in the polymerization temperature of St to 60 °C, which is particularly beneficial in minimizing thermal self-initiation. These findings demonstrate that CPME is a highly promising eco-friendly solvent, offering a viable and effective alternative to hazardous solvents in RAFT polymerization.

In addition to the above-mentioned polar aprotic solvents, EL – a solvent derived from lignocellulosic biomass, has attracted increased attention. It is a protic solvent produced via the fermentation of biomass-derived raw materials (Figure 12). It is known for its low toxicity, biodegradability, high boiling point, and relatively low cost. Moreover, EL is recognized as a safe compound approved by the US Food and Drug Administration for use in pharmaceutical preparations and food additives.^174^

**Figure 12:**
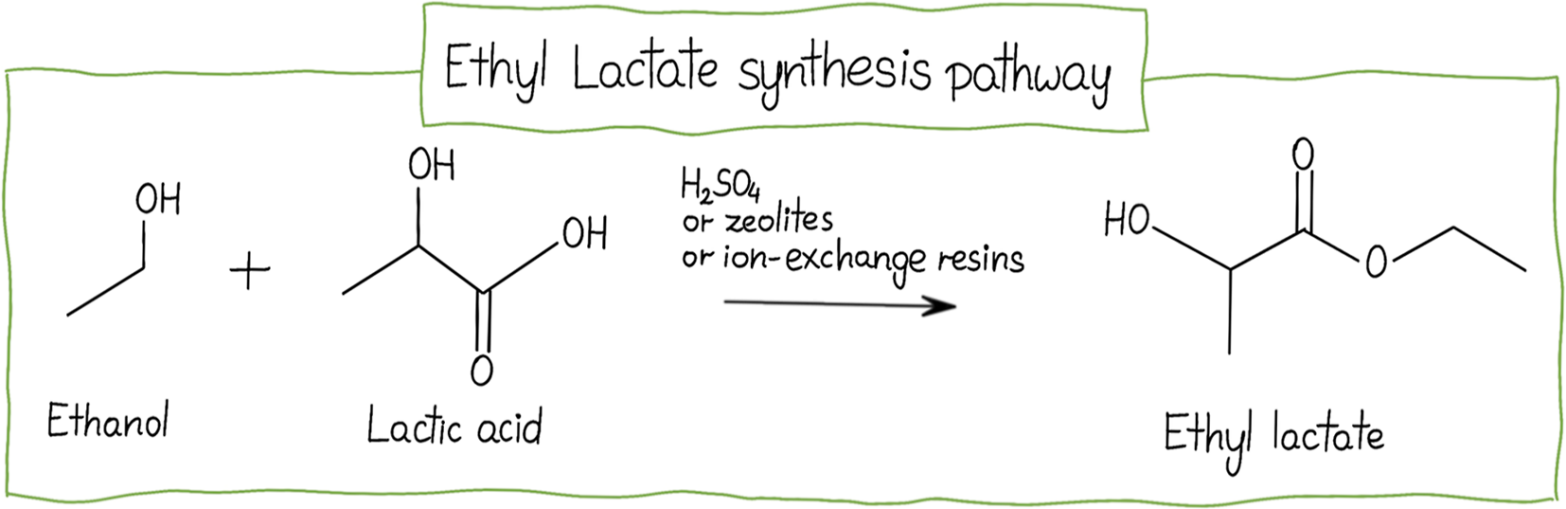
Ethyl lactate synthesis pathway. Adapted with permission from ref 174. Copyright 2011 Royal Society of Chemistry.

The first report demonstrated the suitability of this neoteric solvent for ARGET ATRP of MA.^175^ The solvent showed good solubility for both monomer and polymer. However, all reducing agents, such as ascorbic acid, sodium ascorbate, Na_2_S_2_O_4_, NaHSO_4_, and Na_2_S_2_O_7,_ were partially soluble in EL, which was arguably beneficial to ensure slow feeding of the reducing agent and thus maintain the controlled polymerization. The applied system resulted in well-controlled polymers with a narrow MWD along with moderate monomer conversion (between 70 and 80%), and efficient initiation within 6 h of polymerization. Further studies have shown EL to be an appropriate solvent for the controlled polymerization of a broad spectrum of both hydrophilic and hydrophobic acrylates, including MA, *n*BA, OEGA_480_,^176^ biobased menthyl acrylate (MnA),^177^ and ethyl lactate acrylate (ELA).^178^ The polymerization process of the above-mentioned monomers was conducted using SARA ATRP catalyzed by Cu(0) wire with Me_6_TREN as a ligand.^178^ The polymerization of MA, *n*BA, and OEGA_480_ yielded well-defined polymers with narrow MWD (1.2 for PMA and P*n*BA, 1.29 for POEGA, and a high level of bromine chain-end functionality.^176^ However, in the polymerization of MnA, EL was less efficient than fluorinated alcohol but outperformed EtOH and isopropanol (iPrOH) in terms of conversion and molecular weight control.^177^ Although polymerization in EL achieved high conversion (>80%) and a moderate MWD, notable discrepancies were observed between the theoretical and experimental values. A recent evaluation of biobased ELA produced by SARA ATRP has yielded satisfactory results, demonstrating its potential for future applications. Well-defined polymers with a relatively narrow MWD equal 1.25, and a monomer conversion of 80% were achieved. At the same time the kinetic plot exhibited two distinct linear regimes, suggesting a slight loss of livingness. Furthermore, EL performed slightly worse than other alcohols such as MeOH, EtOH, and TFE. A key improvement was observed with the addition of a small amount of water, which increased the propagation rate, raised monomer conversion to 90%, and produced polymer with lower dispersities below 1.2.^178^ These findings highlight the potential of ethyl lactate as an environmentally friendly solvent, enabling the synthesis of polymers with well-defined architecture and properties.

A recent study further expanded the utility of ethyl lactate by exploring its dual role as both solvent and ligand in the iron-mediated AGET ATRP of MMA.^179^ Although the system produced polymers with controlled molecular weight and preserved chain-end functionality, it required a high catalyst concentration (5,000 ppm), yielded limited conversion (ranging from 31% to 77%), and relatively broad MWD (1.26–1.83), which worsened with increasing concentrations of the reducing agent. Additionally, polymerization was poorly controlled in the later stage. The study also found that increasing the amount of EL led to higher monomer conversion and broader MWD, possibly due to slow deactivation of the growing chains, attributed to the high stability of the deactivator in polar EL. To further investigate EL’s effect on catalyst behavior, other green solvents – ethyl acetate, methyl lactate, and butyl lactate – were tested. As expected, both methyl and butyl lactate exhibited similar metal-coordinating behavior to EL, owing to their O, O-chelating sites.^180^ In contrast, ethyl acetate, when used alone without an external ligand, failed to yield any polymers, excluding its coordinating ability. However, after the addition of an external ligand, polymerization reached higher conversion (74%) and lower dispersity (1.13) than in EL.

The versatility of ethyl lactate as a solvent was also demonstrated in iodide-mediated LRP of various methacrylates, such as MMA, HEMA, PEGMA, [2-(methacryloyloxy)­ethyl]­dimethyl-(3-sulfopropyl)­ammonium hydroxide (SBMA), 2-methacryloyloxyethyl phosphorylcholine (MPC), and 2-methoxyethyl acrylate (MEA), demonstrating good control with polymer molecular weight distribution below 1.3.^181^ Given the use of nontoxic and metabolizable catalysts and biobased solvents such as ethyl lactate, this system shows potential for promising biomedical applications.

In conclusion, the referenced studies collectively illustrate the great potential of ethyl lactate as a “green” solvent for controlled radical polymerization. Its favorable environmental profile and effectiveness across various RDRP methods make it an attractive alternative to conventional, often harmful solvents. Further research into optimizing polymerization conditions in ethyl lactate and expanding its applications in the synthesis of advanced polymeric materials is warranted and may contribute to the development of more sustainable polymer technologies.

### 3.2 Essential Oil-Derived Solvents

An interesting class of biomass-derived solvents includes those obtained from essential oils, particularly terpenes. Notably, the use of terpene-derived solvents in controlled polymerization techniques like ATRP and RAFT remains relatively uncommon. To date, only a few examples have been reported, involving eutectic mixtures composed of menthol, thymol, coumarin, and tetradecanol.^182–185^ These compounds are all naturally sourced from plant-based essential oils, including mint, thyme, citrus fruits, and nutmeg (specifically myristic acid)^186–188^ (see Figure 5).

dl-menthol and tetradecanol have proven effective in the polymerization of hydrophobic monomers, including MA, MMA, St, *n*BA, VC, and VAc by SARA ATRP (at low catalyst loading of 150 ppm) and RAFT techniques. These systems consistently produced well-defined polymers with narrow MWD, less than 1.3, good agreement between theoretical and experimental molecular weights, and preserved chain-end functionality.^182^ In the RAFT polymerization of St and VC, lower monomer conversions (46% and 22%, respectively) were observed, accompanied by significant discrepancies in MWs. Similar issues regarding low conversion were observed in ATRP of St and MMA (40% and 50%, respectively), likely due to reduced solubility of the resulting polymers in EM. Nonetheless, well-defined 4-arm star-shaped PMA was successfully synthesized via SARA ATRP, yielding a narrow MWD of 1.06. Comparative studies evaluated the ATRP of MA in DL-menthol/tetradecanol EM against polymerizations conducted in DMSO, an EtOH/H_2_O mixture (90:10), and the ionic liquid alternative BMIM-PF_6_/DMSO (50:50). Molecular weight control was similar across all solvents. The reaction rate in EM was comparable to EtOH/H_2_O, though slower than in DMSO and BMIM-PF_6_/DMSO. Importantly, at high monomer conversion, phase separation occurred in ATRP of MA using the EM, allowing for straightforward recovery and reuse of both solvent and catalyst. These were successfully reused in subsequent ATRP reactions, achieving results consistent with the initial run. Furthermore, the polymer could be purified via simple vacuum drying, enhancing the method’s sustainability. However, it is worth noting that phase separation may impair EM efficiency in some cases, which warrants further investigation.

Following a similar strategy, a eutectic mixture of l-menthol and thymol was employed for ATRP of both hydrophilic and hydrophobic monomers. Menthol and thymol stand out as significant components of biobased EMs for numerous applications, showcasing their remarkable potential and versatility.^124,189^ Additionally, l-menthol/thymol has a high boiling point and low viscosity, which is advantageous in industrial applications. The possibility of synthesizing various homopolymers with controlled molar masses ranging from 5,000 to 25,000 g/mol, high initiation efficiency, and low dispersity (between 1.02 and 1.47) at low copper catalyst concentration (225 ppm) was demonstrated.^183^ The monomers included hydrophobic MA, MMA, DMAEMA, GMA, and hydrophilic HEA, HEMA, and OEGA_480_. The EM proved miscible with all tested monomers, and no phase separation was observed during polymerization. A key achievement was the “single vessel” synthesis of amphiphilic block copolymers (ABs), such as poly­(2-(dimethylamino)­ethyl methacrylate)-*b*-poly­(2-hydroxyethyl acrylate) (PDAMEMA-*b*-PHEA), PMA-*b*-POEGA, and PMA-*b*-PHEA, using SARA ATRP. This involved sequential monomer addition after high conversion of the first block, without isolating or purifying the macroinitiator. The ability of a single biomass-derived solvent to dissolve both hydrophobic and hydrophilic monomers, along with their resulting polymers, significantly simplifies ABs synthesis compared to other methods that often require multiple steps, protection of functional groups, or toxic solvents.

Building on these results, l-menthol/thymol EMs were successfully applied in ATRP for the synthesis of biocidal polymers based on poly­((methacryloyloxy) ethyl trimethylammonium chloride) (PMETAC) and biobased poly­(thymol methacrylate).^184^ The well-defined polymers were further used to create bioactive coatings aimed at inhibiting bacterial growth and preventing infections.

To expand the utility of natural EM further, RAFT polymerization of *N*-isopropylacrylamide (NIPAM) was investigated in several eutectic mixtures made of thymol/(±)-menthol, tetradecanol/(±)-menthol, and thymol/coumarin.^185^ These systems yielded well-controlled poly­(*N*-isopropylacrylamide) (PNIPAM) with MWDs ranging from 1.18 to 1.34 and monomer conversion above 96%. The polymerization strategy for NIPAM was expanded to include natural deep eutectic monomers (NADEMs), formed by combining NIPAM with natural compounds such as thymol, menthol, coumarin, and tetradecanol. In these systems, NIPAM serves as both monomer and hydrogen bond donor, while the natural compounds act as acceptors. These novel deep eutectic monomers (DEMs) represent a sustainable and low-cost class of materials capable of ultrafast bulk RAFT polymerization, even in the presence of air, without the need for volatile organic solvents. Their nearly 100% atom economy positions them as strong candidates for future large-scale, eco-friendly polymer production.

The flexibility of the described EMs across both ATRP and RAFT systems significantly broadens the scope of copolymer composition. This versatility enables the combination of various monomers and supports the sequential or simultaneous use of ATRP and RAFT techniques, facilitating the synthesis of complex polymer structures with precisely tailored properties and functions.

### 3.3 Vegetable Oil-Derived Solvents

Vegetable oils have been demonstrated to serve as effective, environmentally friendly alternatives to toxic solvents in inverse emulsion systems for both ATRP^108^ and RAFT^107^ polymerization. Several readily available vegetable oils, including avocado, olive, sunflower, rapeseed, rice bran, and grapeseed oils, have been successfully applied as the continuous phase in these systems. Vegetable oils are composed of 94–96% triglycerides, primarily fatty acids and glycerol esters.^190,191^ Their composition varies depending on the type of oil, particularly in terms of saturated and unsaturated fatty acids content. For example, rapeseed oil contains significantly less saturated acids (around 5%), and predominantly oleic acid (∼65%, monounsaturated), and linoleic acid (∼20%, polyunsaturated).^192^ In contrast, sunflower oil contains approximately 15% saturated acids, with 14–43% oleic acid and 44–75% linoleic acid.^193^ Unsaturated fatty acids, especially polyunsaturated, have lower stability compared to their saturated counterparts, making them more susceptible to so-called rancidity – an oxidation process driven by factors such as water, oxygen, metal atoms, or microbial activity.^194^ However, certain oils, such as rapeseed oil, may include antioxidants, for example, vitamin E, which inhibit oxidation, thereby enhancing the stability of the oil. As a result, the best control over HEA polymerization was achieved in rapeseed oil-based emulsion.^108^ Under optimized conditions (see Figure 13), the process yielded high molar mass polymers (*M*_W_ = 230,000 g/mol) with a relatively narrow MWD of 1.43. This marks a significant advancement over analogous syntheses performed in homogeneous aqueous systems, where polymers exhibited broad MWD ranging from 2.49 to 4.29 due to polymer chain coupling processes, as confirmed by bimodal GPC traces.

**Figure 13:**
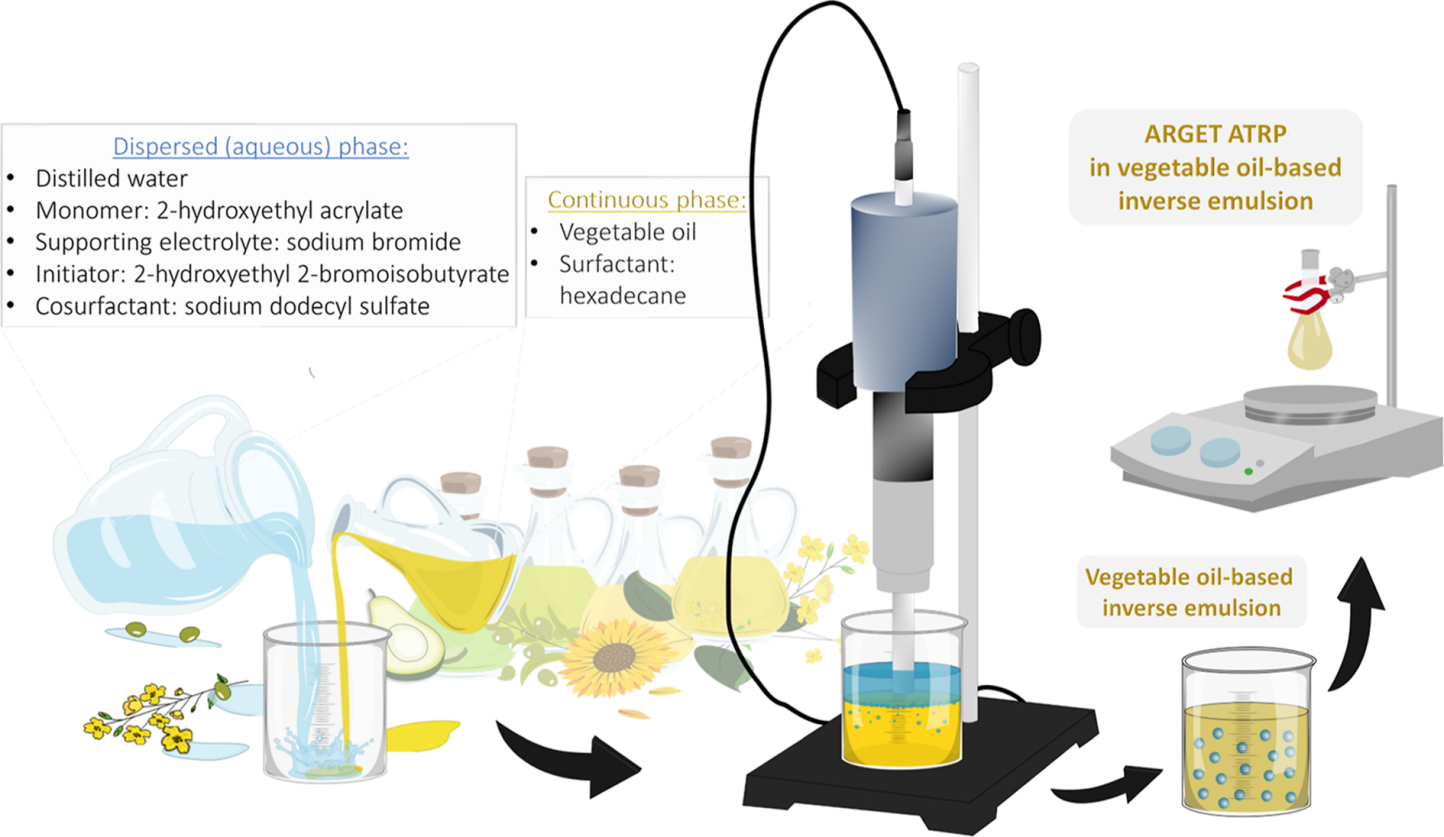
Schematic representation of the reaction environment and conditions for vegetable oil-based inverse emulsion polymerization of HEA. Adopted with permission under a Creative Commons CC-BY 4.0 from ref 108. Copyright 2023 The Authors. Published by the American Chemical Society.

The versatility of this system was further expanded by a chain extension experiment involving the riboflavin-based macroinitiator RF-(POEGMA-Br)_2_ and PHEA, conducted in rapeseed oil-based inverse emulsion. Additionally, least processed oils, such as extra-virgin olive oil and avocado oil, were tested as continuous phases in an inverse emulsion ATRP system.^108^ However, HEA polymerization in these media was less efficient in terms of final product quality and polymerization kinetics, mainly due to limited monomer conversions. This phenomenon is related to the fact that unprocessed oils contain many nutritional compounds, such as phospholipids, α-tocopherol, phenolic compounds, carotenoids, squalene, chlorophyll, and phytosterols. The content of these compounds in extra-virgin oils can adversely affect the polymerization process, e.g., phospholipids can capture metal ions and reduce their catalytic activity.^195^

Beyond ATRP, RAFT photopolymerization in inverse emulsion has also benefited from the use of vegetable oils as alternatives to toxic organic continuous phases in inverse emulsion.^107^ A biocompatible and eco-friendly setup was developed for synthesizing polymeric hollow particles, using an amphiphilic macroRAFT agent that serves as both the initiator and emulsion stabilizer. This RAFT polymerization of ethylene glycol dimethacrylate (EGDMA) was carried out in the presence of sunflower oil, grapeseed oil, and rice bran oil, all of which effectively formed relatively uniform emulsion droplets. While both grapeseed oil and rice bran oil are rich in unsaturated fatty acids, grapeseed oil contains a particularly high concentration of linoleic acid (69.6%).^196^ Rice bran oil also contains significant levels of tocopherol and vitamin E, which possess radical scavenging properties.^197^ Nevertheless, rice bran oil proved efficient for the visible light-induced RAFT polymerization of divinyl monomer at room temperature, resulting in polymer microcapsules with uniform sizes after THF washing.

These approaches highlight the potential of vegetable oils as safe and sustainable alternatives to traditional solvents for synthesizing polymeric microcapsules, particularly for cosmetics, food, and pharmaceutical applications where solvent toxicity is a major concern.

## 4 Summary

This review highlights the critical role of solvents in controlled polymer synthesis and discusses recent advancements in the use of solvents derived from renewable raw materials. Given their influence on reaction kinetics, stability of catalysts, reagent solubility, and the potential for side reactions with monomers or reducing agents, as well as their extensive use, solvents significantly affect not only the overall performance and outcomes of polymerizations but also their cost and environmental footprint. In response to concerns about human and environmental safety, and the reliance on toxic, nonrenewable resources, increasing efforts have been directed toward the use of biomass-derived solvents in RDRP techniques in recent years, see Figure 14.

**Figure 14:**
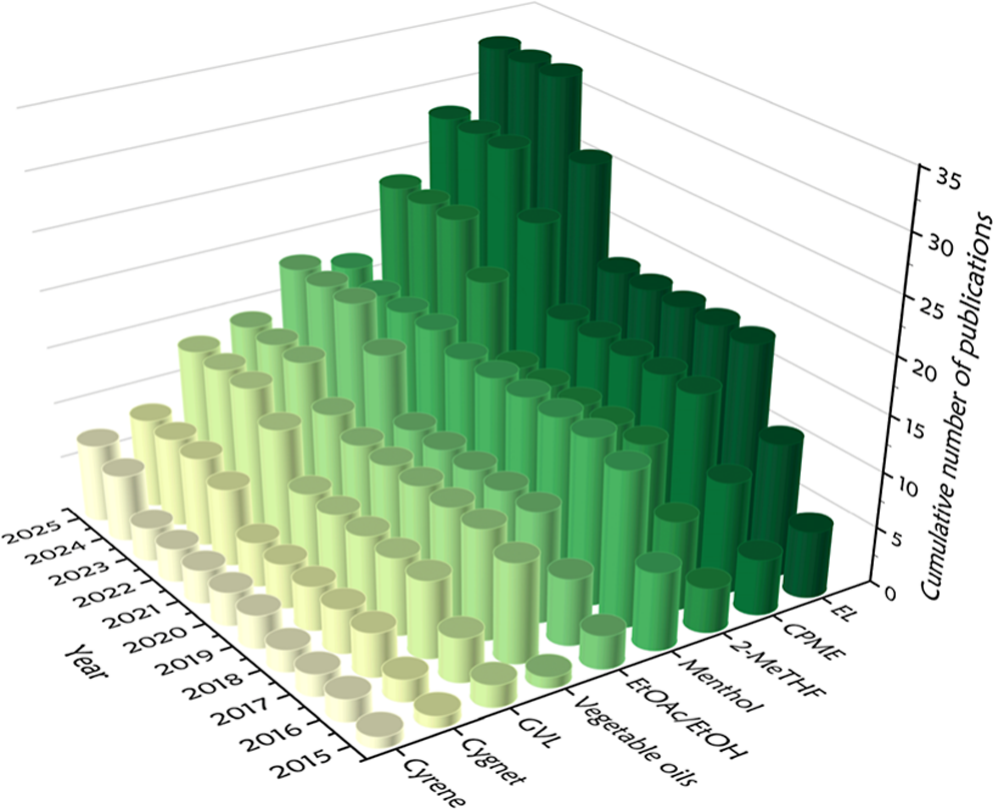
Application of biomass-derived solvents in RDRP.

Introducing alternatives to toxic solvents is one of the first steps toward implementing the principles of green chemistry^198^ to chemical processes. This approach directly supports the principle of “*using renewable raw materials*”, as these solvents are derived from sources such as lignocellulosic biomass, essential oils, or vegetable oils. Additionally, it reduces the use of hazardous chemicals and solvents, thereby improving the overall safety of chemical processes and minimizing the risk of chemical accidents, including spills, explosions, and fires, aligning with the “prevention” principle of green chemistry.

From the perspective of green engineering principles,^199^ biomass-derived solvents offer notable advantages. Their renewability reflects the principle of “*use of renewable rather than depleting resources*”, while their inherently safer nature supports the principle of “*inherent rather than circumstantial*” safety. Together, these attributes contribute to the broader goal of preventing waste generation, rather than relying on downstream treatment.

Although biomass-derived solvents share some physical characteristics with traditional fossil fuel-derived solvents (e.g., flammability), they carry significant advantages, such as sustainable production, lower toxicity, and biodegradability, which translates into potential reductions in waste disposal costs. However, it should be emphasized that the term “biobased solvents” does not always mean “green solvents” – it is crucial to take into account factors such as price, stability, recyclability, types of reagents used, and the overall energy efficiency of their synthesis and modification processes.^200,201^ The use of renewable raw materials does not guarantee an environmentally benign solvent. The thorough analysis should consider all stages of a solvent’s existence – from raw material acquisition, through synthesis, use, to disposal or recycling. For many biomass-derived solvents, even with their advantages in biodegradability and low toxicity, the overall environmental impact may not be entirely favorable, particularly if their production requires toxic reagents, energy-intensive chemical processes, or generates a significant amount of derivative waste.^9,13^ To achieve an accurate evaluation of a solvent’s environmental impact, it is essential to integrate LCA with complementary methodologies. In particular, human toxicity and ecotoxicity represent critical impact parameters,^202,203^ often of greatest concern in the context of occupational exposure or ecological risk arising from solvent waste disposal in landfills.^48^

Despite these environmental benefits, the widespread replacement of toxic solvents with fully ecological alternatives is hindered by their comparatively high cost (see Table 2). The smaller scale of production primarily drives this cost disparity. To overcome this barrier, greater investment and support are needed to scale up production and drive the adoption of these sustainable solutions, contributing to a safer and more environmentally responsible future.

**Table 2:** Cost of Biomass-Derived Replacement for Toxic Solvents^a^

solvent	price ($/L)
eucalyptol	109
ethyl acetate	92
Cyrene	232
Cygnet 0.0	172
PC	85
GVL	128
2-MeTHF	241
CPME	165
cyclopentanone	106
TMO	n.a.
DMI	1,800^b^
NBP	∼740^b^
ethyl lactate (natural)	99
TEGDME	182^b^
l-menthol	155^b^
thymol	75^b^

a Average prices taken from manufacturers’ Web sites; n.a. – data not available.

b Price per 1 KG.

To fully address the use of toxic solvents in RDRP processes, further and more thorough investigations are necessary to explore various alternative approaches and solutions. One key direction is the development of more environmentally friendly production methods for existing biomass-derived solvents. Additionally, there is a need to investigate novel renewable solvents with improved compatibility with catalysts, monomers, and polymers employed in RDRP. The chemical modification of current solvents should also be considered, with a strong emphasis on atom economy, energy saving, and toxicity of reactants used during the process.

Given the importance of life cycle assessment, enhancing solvent recycling and recovery methods is essential. At the same time, greater attention must be paid to the biodegradability and overall environmental impact of biomass-derived solvents at the end of their life cycle. The most favorable option is undoubtedly to reuse the same solvent in subsequent syntheses after simple separation from the reaction mixture, as demonstrated with eutectic mixtures derived from essential oils (see Section 3.2). However, the environmental cost associated with essential oil production must also be considered. Another common method of solvent disposal is incineration, which enables energy recovery and is generally preferable for solvents with low environmental impacts during the production phase. In contrast, for solvents with high production-phase impacts, recovery methods such as distillation are more profitable. Nevertheless, the choice must be made carefully, as the high energy demand of distillation can, in some cases, outweigh its benefits, making incineration the more favorable option.^48,204^

Considering all impact categories outlined in Section 2, the complexity of LCA remains a major challenge, as it involves balancing many competing outcomes. The decision-making process therefore requires further refinement, particularly when upscaling data from the laboratory to the industrial level and when integrating LCA with other risk assessment methods. Only then can establish a transparent and well-defined methodology.^48^

A major step forward in reducing the reliance on petroleum-based products lies in the synthesis of biomass-derived polymers via RDRP techniques in biomass-derived solvents. This approach offers sustainable alternatives to conventional plastics and contributes to lowering the overall environmental footprint.

## Abbreviations

2-Me-THF: 2-methyltetrahydrofuran
ABs: amphiphilic block copolymers
AsAc: ascorbic acid
AsAc-Na: sodium ascorbate
ATRP: atom transfer radical polymerization
BMA: butyl methacrylate
BzA: benzyl acrylate
BzMA: benzyl methacrylate
CL: caprolactone
CPME: cyclopentyl methyl ether
CreMA: cresol methacrylate
CTAs: chain transfer agents
Cu^I^/L: activator complex
Cu^II^/L: deactivator complex
DCM: dichloromethane
DEMs: deep eutectic monomers
DESs: deep eutectic solvents
DMAc: dimethylacetamide
DMAEMA: 2-(dimethylamino)­ethyl methacrylate
DMF: *N*,*N*-dimethylformamide
DMSO: dimethyl sulfoxide
DP: degree of polymerization
*E*: cohesive energy
EBiB: ethyl α-bromoisobutyrate
EBPA: ethyl α-bromophenylacetate
ECiB: ethyl α-chloroisobutyrate
EGDMA: ethylene glycol dimethacrylate
EL: ethyl lactate
ELINCS: European List of Notified Chemical Substances
EMA: ethyl methacrylate
EMs: eutectic mixtures
EtOAc: ethyl acetate
FRP: free radical polymerization
GMA: glycidyl methacrylate
GSK: GlaxoSmithKline
GuMA: guaiacol methacrylate
GVL: γ-valerolactone
H_2_AAIPI: isopropylidene ascorbic acid
HBA: hydrogen bond acceptor
HBD: hydrogen bond donor
HEA: 2-hydroxyethyl acrylate
HEMA: 2-hydroxyethyl methacrylate
HSE: health, safety, and environmental
HSP: Hansen solubility parameters
*I*_eff_: initiation efficiency
Ils: ionic liquids
iPrOH: isopropanol
KAT: Kamlet–Abboud–Taft
*K*_ATRP_: ATRP equilibrium constant
LAc: levulinic acid
LA: lactide
LCA: life cycle analysis
LD_50_: lethal dose
Log *P*: octanol–water coefficient
mPEG: methyl-polyethylene glycol
MA: methyl acrylate
Me_6_TREN: tris­[2-(dimethylamino)­ethyl]­amine
MEA: 2-methoxyethyl acrylate
MMA: methyl methacrylate
MnA: menthyl acrylate
MPC: methacryloyloxyethyl phosphorylcholine
*M*_W_: molecular weight
MWD: molecular weight distribution
NADEMs: natural deep eutectic monomers
nBA: *n*-butyl acrylate
NBP: *N*-butylpyrrolidinone
NIPAM: *N*-isopropylacrylamide
NMP: *N*-methylpyrrolidone
OEGA_480_: poly­(ethylene glycol) methyl ether acrylate
OEGMA: poly­(ethylene glycol) methyl ether methacrylate
PC: propylene carbonate
PDMAEMA: poly­(2-(dimethylamino)­ethyl methacrylate)
PEGMA: poly­(ethylene glycol) methacrylate
PET: photoinduced electron-energy transfer
PHEA: poly­(2-hydroxyethyl acrylate)
PheMA: phenyl methacrylate
PISA: polymerization-induced self-assembly
PMETAC: poly­((methacryloyloxy) ethyl trimethylammonium chloride)
PMA: poly­(methyl acrylate)
PMMA: poly­(methyl methacrylate)
PnBA: poly­(*n*-butyl acrylate)
PNIPAM: poly­(*N*-isopropylacrylamide)
RAFT: reversible addition–fragmentation chain-transfer
RDRP: reversible deactivation radical polymerization
REACH: Registration, Evaluation, Authorization, and Restriction of Chemicals
ROP: ring opening polymerization
SBMA: 2-(methacryloyloxy)­ethyl]­dimethyl-(3-sulfopropyl)­ammonium hydroxide
St: styrene
SyrMA: syringol methacrylate
*t*BA: *tert*-butyl acrylate
TBABr: tetrabutylammonium bromide
TEGDME: tetraethylene glycol dimethyl ether
TFEA: trifluoroethyl acrylate
THF: tetrahydrofuran
ThyMA: thymol methacrylate
TPMA: tris­(2-pyridylmethyl)­amine
TSCA: Toxic Substances Control Act
*V*: molar volume
VAc: vinyl acetate
VaMA: vanillin methacrylate
VC: vinyl chloride
Y-MB: Yamamoto-molecule breaking
γMeMBL: γ-methyl-α-methylene-γ-butyrolactone
δ_D_: dispersion forces
δ_H_: hydrogen bonding ability
δ_P_: polar (dipole–dipole) interactions
